# Systematic benchmarking of single-cell ATAC-sequencing protocols

**DOI:** 10.1038/s41587-023-01881-x

**Published:** 2023-08-03

**Authors:** Florian V. De Rop, Gert Hulselmans, Chris Flerin, Paula Soler-Vila, Albert Rafels, Valerie Christiaens, Carmen Bravo González-Blas, Domenica Marchese, Ginevra Caratù, Suresh Poovathingal, Orit Rozenblatt-Rosen, Michael Slyper, Wendy Luo, Christoph Muus, Fabiana Duarte, Rojesh Shrestha, S. Tansu Bagdatli, M. Ryan Corces, Lira Mamanova, Andrew Knights, Kerstin B. Meyer, Ryan Mulqueen, Akram Taherinasab, Patrick Maschmeyer, Jörn Pezoldt, Camille Lucie Germaine Lambert, Marta Iglesias, Sebastián R. Najle, Zain Y. Dossani, Luciano G. Martelotto, Zach Burkett, Ronald Lebofsky, José Ignacio Martin-Subero, Satish Pillai, Arnau Sebé-Pedrós, Bart Deplancke, Sarah A. Teichmann, Leif S. Ludwig, Theodore P. Braun, Andrew C. Adey, William J. Greenleaf, Jason D. Buenrostro, Aviv Regev, Stein Aerts, Holger Heyn

**Affiliations:** 1 VIB Center for Brain and Disease Research, Leuven, Belgium; 2 Department of Human Genetics, KU Leuven, Leuven, Belgium; 3 Institut d’Investigacions Biomèdiques August Pi i Sunyer (IDIBAPS), Barcelona, Spain; 4 CNAG-CRG, Centre for Genomic Regulation (CRG), Barcelona Institute of Science and Technology (BIST), Barcelona, Spain; 5 Klarman Cell Observatory, Broad Institute of MIT and Harvard, Cambridge, MA USA; 6 Broad Institute of MIT and Harvard, Cambridge, MA USA; 7 Department of Stem Cell and Regenerative Biology, Harvard University, Cambridge, MA USA; 8 Gene Regulation Observatory, Broad Institute of MIT and Harvard, Cambridge, MA USA; 9 Department of Genetics, Stanford University, Stanford, CA USA; 10 Gladstone Institute of Neurological Disease, San Francisco, CA USA; 11 Wellcome Sanger Institute, Cambridge, UK; 12 Department of Molecular and Medical Genetics, Oregon Health & Science University, Portland, OR USA; 13 Division of Hematology & Medical Oncology, Knight Cancer Institute, Oregon Health & Sciences University, Portland, OR USA; 14 Division of Oncologic Sciences, Knight Cancer Institute, Oregon Health & Sciences University, Portland, OR USA; 15 Berlin Institute of Health at Charité–Universitätsmedizin Berlin, Berlin, Germany; 16 Max Delbrück Center for Molecular Medicine in the Helmholtz Association (MDC), Berlin Institute for Medical Systems Biology (BIMSB), Berlin, Germany; 17 Laboratory of Systems Biology and Genetics, Institute of Bioengineering, School of Life Sciences, École Polytechnique Fédérale de Lausanne (EPFL), Lausanne, Switzerland; 18 Swiss Institute of Bioinformatics (SIB), Lausanne, Switzerland; 19 Universitat Pompeu Fabra (UPF), Barcelona, Spain; 20 Vitalant Research Institute, San Francisco, CA USA; 21 Department of Laboratory Medicine, University of California, San Francisco, CA USA; 22 Adelaide Centre for Epigenetics and the South Australian Immunogenomics Cancer Institute, Faculty of Health and Medical Sciences, The University of Adelaide, Adelaide, South Australia Australia; 23 University of Melbourne Centre for Cancer Research, Victoria Comprehensive Cancer Centre, Melbourne, Victoria Australia; 24 Digital Biology Group, Bio-Rad, Pleasanton, CA USA; 25 Departament de Fonaments Clínics, Facultat de Medicina, Universitat de Barcelona, Barcelona, Spain; 26 Centro de Investigación Biomédica en Red de Cáncer (CIBERONC), Madrid, Spain; 27 Institució Catalana de Recerca i Estudis Avançats (ICREA), Barcelona, Spain; 28 ICREA, Barcelona, Spain; 29 Department of Physics/Cavendish Laboratory, University of Cambridge, Cambridge, UK; 30 Chan Zuckerberg Biohub, San Francisco, CA USA; 31 Koch Institute of Integrative Cancer Research, Cambridge, MA USA; 32 Howard Hughes Medical Institute, Department of Biology, Massachusetts Institute of Technology (MIT), Cambridge, MA USA

**Keywords:** Data processing, Epigenetics

## Abstract

Single-cell assay for transposase-accessible chromatin by sequencing (scATAC-seq) has emerged as a powerful tool for dissecting regulatory landscapes and cellular heterogeneity. However, an exploration of systemic biases among scATAC-seq technologies has remained absent. In this study, we benchmark the performance of eight scATAC-seq methods across 47 experiments using human peripheral blood mononuclear cells (PBMCs) as a reference sample and develop PUMATAC, a universal preprocessing pipeline, to handle the various sequencing data formats. Our analyses reveal significant differences in sequencing library complexity and tagmentation specificity, which impact cell-type annotation, genotype demultiplexing, peak calling, differential region accessibility and transcription factor motif enrichment. Our findings underscore the importance of sample extraction, method selection, data processing and total cost of experiments, offering valuable guidance for future research. Finally, our data and analysis pipeline encompasses 169,000 PBMC scATAC-seq profiles and a best practices code repository for scATAC-seq data analysis, which are freely available to extend this benchmarking effort to future protocols.

## Abstract (web summary)

Benchmarking of scATAC-seq protocols demonstrates the effects of data quality on downstream analyses.

## Main

Data quality in single-cell sequencing studies directly influences successful interpretation. Technologies that generate high and accurate molecule counts allow for a precise characterization of cells and can yield deep insights into the underlying tissue biology. To facilitate an informed decision-making process on the choice of technology, systematic benchmarking efforts have been performed for sample preparation protocols^1^ and single-cell RNA-sequencing (scRNA-seq) technologies^2^. However, such efforts are still lacking for chromatin accessibility profiling technologies^3^, such as single-cell assay for transposase-accessible chromatin by sequencing (scATAC-seq)^4^. Recent technological advances enable large-scale studies and have established scATAC-seq as a major pillar in systematic profiling efforts^5–7^. While transcriptomic profiles based on scRNA-seq provide information to infer cellular phenotypes, scATAC-seq detects accessible chromatin sites that pinpoint genomic regions involved in gene regulation. The latter is particularly important to derive mechanistic insights into cell-type development and differentiation or to identify drivers of cell state dynamics following a stimulus, perturbations or disease (Table 1).

**Table 1:** Estimated costs per experiment of 5,000 cells

	10x v2	10x multiome	Bio-Rad ddSEQ	s3-ATAC	HyDrop
Sequenced reads per cell at saturation	55,000	68,000	19,000	1,467,000	10,000
Expected unique fragments per cell	22,427	10,155	5,249	66,130	1,884
Expected unique fragments in peaks per cell	13,680	6,398	2,992	12,565	716
Assay price per 5,000 cells	$1,565	$2,843	$1,100	$800	$100
Sequencing cost	$791	$978	$273	$21,088	$144
Total cost per cell	$0.471	$0.764	$0.275	$3.80	$0.049

Saturation sequencing depth is defined as the depth at which 50% of fragments in cells are duplicates. The expected number of unique fragments was calculated as the expected number of unique fragments at saturation sequencing depth, interpolated or extrapolated using a Langmuir model, and multiplied by the median FRIP score per technique to achieve the expected in peaks count. The price per 5,000 cells is for one 10x lane, one ddSEQ lane, four s3-ATAC plates (1,440 cells each, for 5,760 cells in total) or one HyDrop ATAC run. Sequencing cost is defined as the price of sequencing 5,000 cells to saturation depth, calculated at a cost of $2.875 per 1 million reads, using NovaSeq S2 100 cycles (10x v2, 10x multiome and HyDrop) or 200 cycles (ddSEQ and s3-ATAC; 4,000 million reads sequenced at $10,000 to $11,000 total cost). The sequencing cost for 10x multiome RNA component was not included.

This work benchmarked eight scATAC-seq methods across 47 experiments, including technical and center replicates for Bio-Rad ddSEQ, HyDrop, s3-ATAC and different variants of the 10x Genomics scATAC-seq assay. Peripheral blood mononuclear cells (PBMCs) served as a reference sample to minimize technical variability related to sample preparation, allowing the systematic evaluation of method performance across multiple quality control metrics. PUMATAC^8^, our pipeline for universal mapping of ATAC-seq data, further reduced variability in data preprocessing and enabled systematic benchmarking of the presented data, also allowing the extension to future technologies. Differences between methods were driven by sequencing library complexity and tagmentation specificity, with consequences on the performance of key features for data analysis and interpretation and the integration of datasets into joint cellular atlases.

## Results

**Fig. 1:**
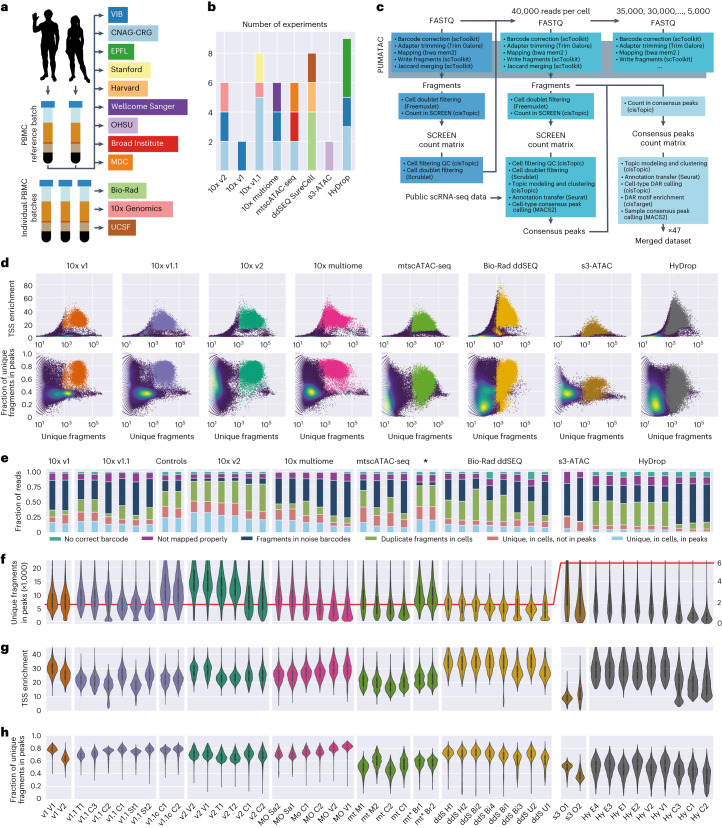
Overview of experimental design and low-level quality control metrics. **a**, Schematic overview of the experimental design; CNAG-CRG, Centro Nacional de Análisis Genómico; EPFL, École Polytechnique Fédérale de Lausanne; OHSU, Oregon Health & Science University; MDC, Max Delbrück Center for Molecular Medicine in the Helmholtz Association; UCSF, University of California San Francisco; VIB, Vlaams Instituut voor Biotechnologie. **b**, Bar chart of the number of experiments performed per technology colored by institute of origin. **c**, Diagram of the universal PUMATAC data analysis pipeline and further downstream analyses; QC, quality control. **d**, Distribution of TSS enrichment, FRIP and total unique fragment counts for all barcodes across all technologies. The blue, green and yellow color scale denotes local density. Saturated colors mark barcodes identified as cells. The distributions for individual samples are shown in Extended Data Fig. 2. **e**, Stacked bar plot showing the fraction of reads lost across each step of data processing. ‘Unique, in cells, in peaks’ is the final fraction of sequencing reads retained in count matrices. Asterisk among technology names indicates mtscATAC-seq samples performed on PBMC that were viability FAC-sorted prior to tagmentation. **f**–**h**, Distributions of unique fragments in peaks (**f**), TSS enrichment (**g**) and fraction of unique fragments in peaks (**h**) in filtered cell barcodes. The scale was shifted to accommodate lower fragment counts in s3-ATAC and HyDrop, indicated by a red line denoting a value of 6,000; *n* = 178,453 cells (before doublet filtering) examined over 47 independent experiments. Median values are indicated by central white dots, quartiles are indicated by black boxes, and minima/maxima/centers are not indicated. Source data

We performed a systematic, multicenter benchmarking study of eight different scATAC-seq protocols. Our benchmark includes all variants of 10x Genomics scATAC-seq (v1 (ref. ^9^), v1.1, v2, multiome and mitochondrial scATAC (mtscATAC)^10^) as well as Bio-Rad ddSEQ^11^, HyDrop^12^ and s3-ATAC^13^. A reference sample of PBMCs from two adult donors (male and female) mixed at a 1:1 ratio was used to simulate complex sample composition (containing multiple cell types and conditions) and minimize sample preparation complexity. This reference sample was distributed for a multicenter benchmarking study and was used for all experiments, with the exception of the indicated replicate datasets (Fig. 1a). Each experiment was performed in technical replicates across centers with a target of 3,000 cells per sample to recover all major PBMC cell types, such as T and B cell subtypes, natural killer (NK) cells, monocytes and dendritic cells (DCs). In total, we generated 47 datasets (Fig. 1b), including replicates across at least three centers with two technical replicate experiments for all methods, except s3-ATAC and 10x v1.

### PUMATAC: a generic and automated analysis pipeline

To compare different protocols in a unified manner, all sequencing data were analyzed using PUMATAC (pipeline for universal mapping of ATAC-seq data)^8^, a newly developed scATAC-seq preprocessing pipeline (Fig. 1c). Briefly, PUMATAC takes scATAC-seq data and applies a set of uniform preprocessing steps, including cell barcode error correction, adapter trimming, reference genome alignment and mapping quality filtering (Methods). We chose bwa-mem2 for read alignment as bwa-mem was used in the original manuscripts describing the eight technologies. PUMATAC then records aligned chromatin fragments in the ubiquitous bed-like ‘fragments file’ format, a tab-separated text file providing the start and end positions of each fragment and its corresponding cell barcode. On average, across samples, 97% of aligned fragments by PUMATAC shared their barcodes and coordinates with their counterparts aligned by CellRanger (10x Genomics; Supplementary Fig. 1a). PUMATAC currently includes workflows for the eight technologies described in this benchmark study but allows for the modular addition of new scATAC-seq sequencing methods by modifying existing templates^14^. PUMATAC also features a reimplementation of bap2 (ref. ^15^), which detects and merges barcodes with significant fragment identity overlap. This step is necessary to merge bead doublets (inherent to Bio-Rad ddSEQ data) and was performed on all techniques for uniformity (Supplementary Fig. 1c). As expected, between 55% and 92% of Bio-Rad-filtered cell barcodes were the result of such merging events due to the method’s bead overloading strategy, as opposed to a median of 1.5% for non-Bio-Rad samples (Supplementary Fig. 1b).

After preprocessing, the fragments files were postprocessed using cisTopic^16^. High-quality cells were separated from background noise barcodes and low-quality cells using sample-specific, algorithmically defined minimum thresholds on the number of unique fragments and transcription start site (TSS) enrichment (Fig. 1d, Extended Data Fig. 2 and Methods). Background noise barcodes are barcodes enriched with trace amounts of fragments through three mechanisms: (1) ambient accessible chromatin fragments may initiate barcoding reactions inside cell-free droplets, (2) unbound barcodes in the bead stock solutions may contaminate legitimate barcoding reactions, or (3) barcode impurities on the beads may lead to ambivalent labeling of chromatin fragments from a single nucleus^15^. Nucleosome-free fragments from viable cells show enrichment around TSSs, a hallmark used to exclude low-quality cells from subsequent analysis^4,17^. All original sequencing datasets were then downsampled to 40,796 reads per cell, the highest common number of reads available across all samples (hereafter referred to as the 40k dataset). Finally, the downsampled datasets were processed again using PUMATAC and cisTopic and subset to the original set of filtered cell barcodes, ensuring that the number of reads per cell remained constant. All downstream analyses were performed on the 40k dataset or further downsampled sets derived thereof.

To generate a preliminary count matrix, fragments were first counted in the ENCODE SCREEN regions^18^. Cell doublets were detected and removed based on cell-type identity using Scrublet^19^ and donor genotype identity using Freemuxlet^20^ (Methods). Next, cells were clustered using cisTopic and annotated through an automated label transfer from independently annotated scRNA-seq PBMC reference data^21^ using Seurat^22^. For each sample, cell-type-specific chromatin accessibility peaks were detected using MACS2 (ref. ^23^) and aggregated into a high-quality set of sample-specific consensus peaks. This strategy was used to discover peaks specific to smaller cell populations. Each sample’s fragments were then recounted in its own consensus peak set, generating a consensus peak count matrix before a second round of cisTopic clustering and Seurat label transfer was performed. The resulting 47 annotated and clustered datasets were used for all downstream analyses and comparisons. Finally, a third round of consensus peak sets was generated from all samples and aggregated into one master set of PBMC consensus peaks, which was used to generate a merged count matrix containing cells from all experiments. This strategy was used to generate three distinct benchmarking datasets containing (1) all cells at original sequencing depth (Extended Data Fig. 3), (2) all cells at 40k sequencing depth (Fig. 4b) and (3) an equal number of cells per cell type at 40k sequencing depth (Extended Data Fig. 4a). We also generated randomly sampled sets (2,500, 2,000, 1,500, 1,000 and 500 cells) to investigate potential effects of total cell counts on label transfer and differentially accessible region (DAR) calling. All merged benchmarking datasets and all individual samples are available in SCope, our public single-cell viewer (https://scope.aertslab.org/#/scATAC-seq_Benchmark/scATAC-seq_Benchmark/welcome).

### Chromatin fragment capture and sequencing efficiency

The PUMATAC analysis workflow discarded sequencing reads at several filtering steps throughout preprocessing. The fraction of reads that ultimately remains in the filtered count matrix is an important measure of assay quality as it is inversely related to sequencing costs. Furthermore, understanding the causes of read loss can shed light on how such losses affect downstream analyses and how to improve reaction chemistries or sample preparation workflows. We therefore calculated the fraction of total reads lost at each filtering stage and quantified differences in these metrics between samples (Fig. 1e). The first loss occurred when barcode reads were corrected based on a barcode sequence whitelist and when mapped to the reference genome. More specifically, reads without a valid cell barcode or those that could not be aligned with a Phred mapping quality score of >30 were discarded. Both steps combined accounted for relatively small losses, ranging from 10.4% for 10x v2 to 22.7% for HyDrop. Filtering true cells from background noise and low-quality cells resulted in significantly larger losses for some methods. Between 7% (10x v2) and 60% (s3-ATAC) of mapped fragments were discarded at this stage. Notably, fluorescence-activated cell sorting (FACS) of live cells before nuclei extraction reduced such losses to below 6% for two mtscATAC-seq experiments (samples mt* Br1 and mt* Br2) compared to 36% in mtscATAC-seq without FACS (samples mt M1, mt M2, mt C1 and mt C2), suggesting that the additional FACS step removes significant amounts of ambient chromatin and damaged cells. Because diploid cells can produce a maximum of two unique fragments originating from a given accessible region and the fact that scATAC-seq data are sparse compared to all possible accessible sites, accessibility in peak regions is usually binarized within a single cell. Out of the quality-filtered fragments, a large fraction includes duplicates resulting from fragment amplification steps during sample preparation, which are discarded instead of quantified. The fraction of duplicate reads in our datasets ranged from 5% for s3-ATAC to more than 70% for HyDrop when sequenced at 40,000 reads per cell. Finally, a significant portion of unique fragments in each cell did not overlap with peak regions and was thus not considered in analysis methods based on peak count matrices. The proportion of such fragments varied between 39% (10x v2) and 82% (s3-ATAC) of all unique reads in cells. As a consequence of the above filtering steps, the fraction of original sequencing reads that are ultimately associated with cells, not duplicated and located within peak regions, can be surprisingly low, ranging between 28% (10x v2) and below 4% (s3-ATAC and HyDrop). These findings suggest that sequencing scATAC-seq experiments are generally highly inefficient, and protocol optimization steps should be performed to maximize cell quality and library complexity and minimize ambient chromatin contamination and PCR duplication. While both s3-ATAC and HyDrop attain a markedly lower sequencing efficiency than commercial assays, this reduced sensitivity was caused by different mechanisms; s3-ATAC samples contained many fragments outside of peak regions, whereas HyDrop fragments were highly duplicated.

### Sensitivity and specificity

**Extended Data Fig. 1:**
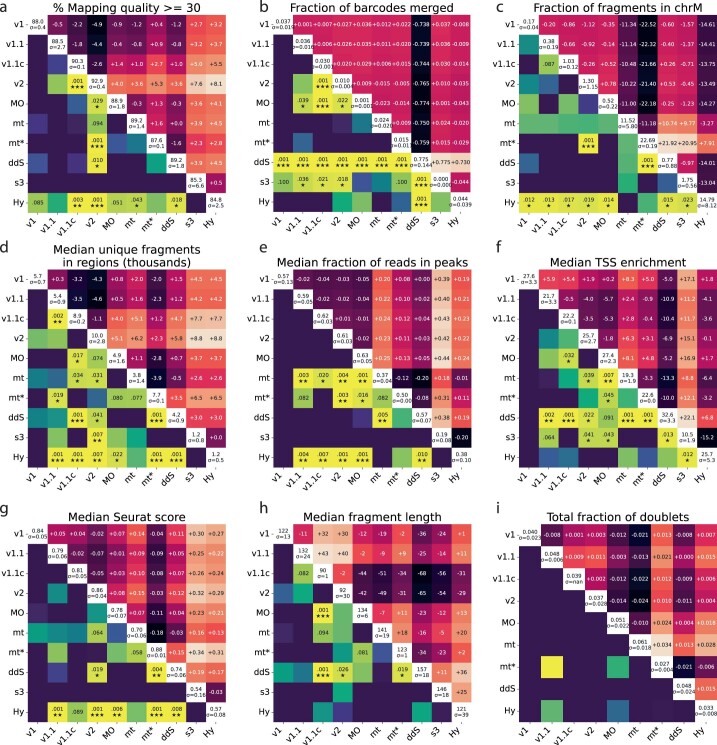
Games-Howell heatmaps of several quality control metrics. Diagonal shows mean values across samples from each technology. Upper triangle shows pairwise differences between means. Lower triangle indicates significance of expected pairwise differences. mt* indicates FAC sorting for these mtscATAC-seq samples. **a**, % of all reads with Phred mapping quality score >= 30. **b**, Fraction of barcodes being the result of a Jaccard merging event (= being a barcode multiplet). **c**, Fraction of fragments aligned to mitochondrial genome. Figures d-g are cell-level statistics, meaning first a median is taken within each sample’s cells, and these medians are compared between techniques. **d**, Median unique fragments in peaks, in cells (x1000). **e**, Median fraction of reads in peaks in cells. **f**, Median TSS enrichments of reads within cells. **g**, Median Seurat scores across cells. **h**, Median fragment length in fragments files. **i**, Total number of doublets (both Freemuxlet + Scrublet-identified) filtered out, expressed as a fraction of total cells before doublet filtering. Source data

After initial filtering steps, the remaining cells exhibited stark differences in quality metrics across techniques. In terms of sensitivity, 10x v2 performed best, recovering, on average, 10,021 unique fragments in peaks per cell, which was significantly higher than Bio-Rad ddSEQ (4,228), HyDrop (1,180) and s3-ATAC (1,203; Fig. 1f and Extended Data Fig. 1d). TSS enrichment was also significantly stratified across methods with 10x v1.1, mtscATAC and s3-ATAC scoring 21.7 or lower, 10x v1, v2, multiome and HyDrop samples scoring between 25.2 and 27.6 and Bio-Rad ddSEQ scoring, on average, 32.6 (Fig. 1g and Extended Data Fig. 1f). The fraction of reads in peaks (FRIP) was significantly lower in HyDrop (38.5%), mtscATAC-seq (37.3%) and s3-ATAC (18.9%) than in Bio-Rad ddSEQ and the other 10x methods (57.3–62.7%; Fig. 1h and Extended Data Fig. 1e). Whereas the same method of nuclei extraction was used for all 10x and HyDrop samples (except for mtscATAC where Tween 20 and digitonin were omitted from the cell lysis buffer to retain mitochondrial chromatin), Bio-Rad ddSEQ and s3-ATAC experiments used a lysis buffer without NP-40 or Dounce homogenization in NIB-HEPES with 0.1% Tween and 0.1% NP-40 to extract nuclei, respectively. To investigate whether differences in FRIP, TSS enrichment or number of unique fragments between 10x/HyDrop, Bio-Rad ddSEQ and s3-ATAC could be attributed to differences in nuclei extraction, we performed the following three additional 10x v1.1 experiments: (1) in control experiment 1 (sample 10x v1.1c C1), we used a custom lysis buffer without NP-40 (as in Bio-Rad ddSEQ); (2) in control experiment 2 (sample 10x v1.1c C2), we extracted nuclei using Dounce homogenization (as described in s3-ATAC); and (3) in control experiment 3 (sample 10x v1.1 C3), we used the standard 10x v1.1 nuclei extraction protocol. Both control runs (C1 and C2) generated cells with FRIP and TSS enrichment scores on par with standard 10x v1.1 runs but retrieved a higher than average number of unique fragments. This suggests that the nuclei extraction method was not the sole driving factor causing reduced performance (for example, fragment numbers, FRIP and TSS enrichment) in Bio-Rad ddSEQ and s3-ATAC samples. Finally, we found that Bio-Rad ddSEQ samples had a higher median fragment length than all other techniques (Extended Data Fig. 1h).

**Fig. 2:**
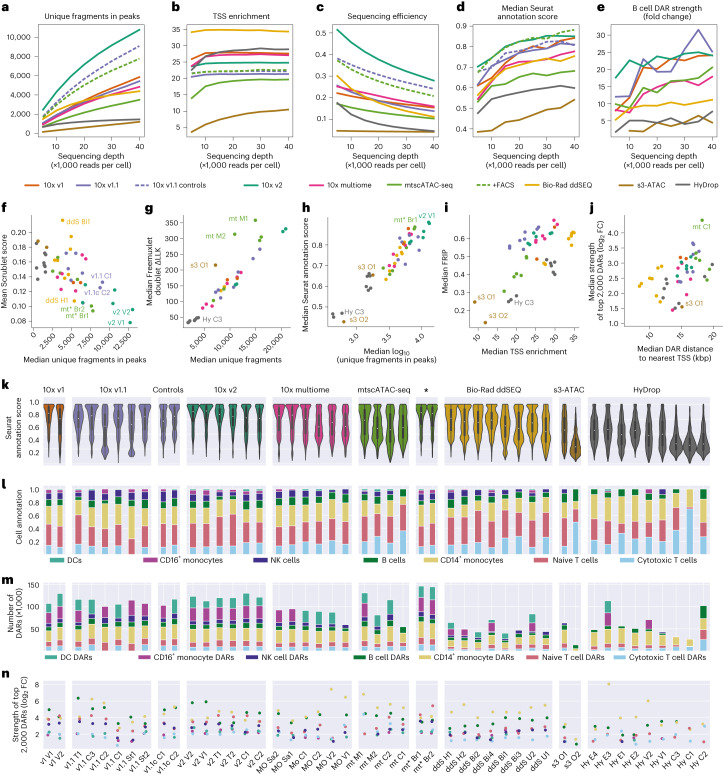
Differences in automated cell-type annotation accuracy and differential region calling between techniques. **a**–**e**, Line graphs showing the effect of sequencing depth (×1,000 reads per cell) on unique fragments in peak regions (**a**), TSS enrichment (**b**), sequencing efficiency (fraction of reads that are associated with filtered cells, not duplicated and located within a peak region; **c**), median Seurat label transfer score (**d**) and fold change enrichment of B cell DARs (**e**). **f**, Scatter plot of mean Scrublet score and median unique number of fragments in peaks across cells. **g**, Scatter plot of the median LLK (log likelihood) of Freemuxlet’s per cell doublet classification and median unique number of fragments across cells. **h**, Scatter plot of median Seurat score and median log_10_ of unique fragments in peaks in cells. **i**, Scatter plot of median FRIP and median TSS enrichment across cells in each sample. **j**, Scatter plot of log_2_ (fold change) (log_2_ (FC)) enrichment of the top 2,000 DARs across cell types and median distance of DARs to the nearest TSS (bp). Sample s3 O2 is omitted due to it being an outlier with a median distance of >300,000 bp; kbp, kilobase pairs. In **f**–**j**, each point represents one experiment, and points are colored by technique. **k**, Distributions of Seurat scores across samples. **l**, Fraction of cell types recovered in cells from individual samples; *n* = 169,156 cells (after doublet filtering) examined over 47 independent experiments. Median values are indicated by central white dots, quartiles are indicated by black boxes, and minima/maxima/centers are not indicated. **m**, Number of DARs recovered per cell type across samples. **n**, log_2_ (fold change enrichment) of the top 2,000 DARs colored by cell type. Source data

With increasing sequencing depth, the number of unique fragments increased but saturated at different levels across techniques. For example, at lower sequencing depths, ddSEQ samples performed better than 10x v1, v1.1 and multiome, an effect that was reversed at a higher sequencing depth (Fig. 2a). TSS enrichment was also dependent on sequencing depth but saturated rapidly (Fig. 2b). Sequencing efficiency decreased with higher sequencing depths due to the increase in duplicate reads (Fig. 2c).

We also investigated the performance of the scRNA-seq component of the six 10x multiome experiments. Therefore, we first analyzed the unfiltered gene expression count matrices to define optimal thresholds of unique molecular identifiers (UMIs) for cell filtering (Extended Data Fig. 5a). We then downsampled the RNA-seq data to a common depth (28,417 reads per cell), realigned the downsampled data and compared the number of genes and unique fragments in peaks in the superset of barcodes identified in either the chromatin accessibility or gene expression component (Extended Data Fig. 5b). In four of six samples, scRNA-seq and scATAC-seq components correlated strongly and identified the same barcodes. However, in two replicates (samples MO C1 and MO C2), almost 4,000 barcodes were exclusively identified in the RNA component (Extended Data Fig. 5c), suggesting that cell filtering based on both scRNA-seq and scATAC-seq metrics can generate false-negative cell calls in at least one modality.

### Doublet counts and cell-type label transfer efficiency

We identified doublets (two cells with the same cell barcode) using simulated data or genotype information with Scrublet and Freemuxlet, respectively (Methods). Scrublet’s doublet score linearly decreased with median unique fragments in peaks (Fig. 2f), and Freemuxlet’s confidence (that is, difference in log likelihood between doublet and singlet assignments) increased with these metrics (Fig. 2g), suggesting that the number of fragments is a critical factor behind doublet detection. Outlier results for s3-ATAC O1, mtscATAC M1 and mtscATAC M2 could be explained by the increased read lengths (and thus increased single-nucleotide polymorphism (SNP) recovery) of 74 base pairs (bp) and 150 bp, respectively, as opposed to 50 bp for the other samples.

Next, we annotated cell types by inferring gene activities using chromatin accessibility in a genomic window around the TSS and subsequent label transfer from annotated scRNA-seq reference datasets using Seurat^22^. When assigning cell-type annotations, Seurat returns a confidence score to each assignment representing the majority percentage of a weighted vote based on cell types present in the reference. On average, a Seurat label transfer score of 0.7 corresponded to an 82% concordance between cell-type identities assigned to ATAC and RNA components of multiome samples, although this effect was cell-type dependent (Extended Data Fig. 6a,b). Similar to Scrublet and Freemuxlet, Seurat’s confidence was strongly dependent on sequencing depth (Fig. 2d and Extended Data Fig. 7a) and the number of unique fragments per cell (Fig. 2h), confirming the number of fragments to be a strong predictor for the quality of downstream analyses. Consequently, both 10x and Bio-Rad ddSEQ methods attained high median label transfer scores, while HyDrop and s3-ATAC scored markedly lower (Fig. 2k and Extended Data Fig. 1g). The most common PBMC types, including B cells, T cells and CD14^+^ monocytes, were recovered by all techniques. However, the differences between methods became evident when focusing on rare cell types, such as DCs and NK cells, showing the lowest assignment scores across techniques (Fig. 2l and Extended Data Fig. 6). For the latter cell types, s3-ATAC and HyDrop recovered a lower fraction or no cells, suggesting that increased sensitivity is required for comprehensive cell-type annotation. Following cell-type assignment, we aggregated high-quality cell-type-specific peaks into consensus peaks. Across all samples and techniques, the fraction of reads within these consensus peaks was strongly correlated with TSS enrichment, affirming that TSS enrichment can be used as a predictor of signal-to-noise ratio before and independent of peak calling (Fig. 2i).

### DARs

Similar to scRNA-seq, scATAC-seq data interpretation largely relies on the identification of signature features between cell types and states. Therefore, we calculated DARs between cell types within each of the 47 samples and evaluated each method’s performance by comparing the number and median fold enrichment strength of DARs recovered across methods. These metrics strongly varied by cell type. In CD14^+^ monocytes, a cell type that was identified in all 47 samples, 10x methods recovered between 26,000 and 30,000 DARs and HyDrop recovered 29,000, whereas s3-ATAC and ddSEQ only recovered 17,000 and 15,000, respectively (Fig. 2m). A similar contrast was observed in the strength of these DARs; the top 2,000 CD14^+^ monocyte DARs recovered by 10x methods were a median of 17.7- to 23.5-fold enriched in CD14^+^ monocytes compared to the other cell types, while this fold enrichment was 10.6-fold, 7.5-fold and 4.9-fold in ddSEQ, HyDrop and s3-ATAC, respectively (Fig. 2n). Of note, DAR strength was positively dependent on sequencing depth (Fig. 2e and Extended Data Fig. 7c), while the number of DARs recovered was not (Extended Data Fig. 7b).

**Fig. 3:**
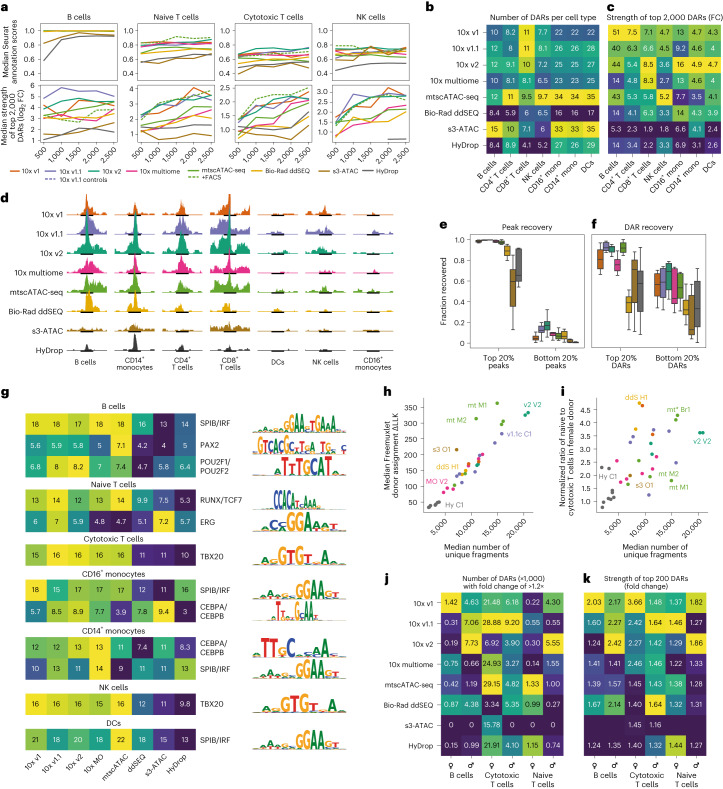
Performance differences in detection of motif enrichment and sexual dimorphisms. **a**, Dependency of Seurat label transfer scores and average log_2_ (fold change) of the top 2,000 DARs in selected cell types on the total number of cells in selected cell types. **b**,**c**, Heat map of the number of DARs (**b**) and heat map of the fold change enrichment (**c**) of the top 2,000 DARs sourced from the cell-type fair merged sets for every technique across cell types. Colors are scaled per column; mono, monocytes. **d**, Fragment coverage within each cell type’s strongest common DAR found in the merged set. Each cell type contains an equal number of cells across technologies. All tracks are scaled to the same absolute coverage. The 10x v1 track is slightly truncated to accommodate **j**. **e**,**f**, Fraction of the top 20% and bottom 20% peaks and DARs found by the merged cell-type fair set recovered in subsets from individual technologies. **g**, Heat map of the normalized enrichment score of cell-type-specific transcription factor motifs. Colors are scaled per row. **h**, Scatter plot of median Freemuxlet donor assignment log likelihood difference to second-best guess and median number of unique fragments. **i**, Scatter plot of the ratio of naive T cells to cytotoxic T cells in male and female subpopulations and median number of unique fragments. In **h** and **i**, each point represents one experiment, and points are colored by technique. **j**, Heat map of the number of sex-specific DARs. Colors are scaled per column. **k**, Heat map of fold change enrichment of the 200 strongest sex-specific DARs. Colors are scaled per column. Source data

Total cell counts and cell counts within each cell type were not equal across all 47 samples (Fig. 2l and Supplementary Fig. 2c). To investigate the effect of total cell count on Seurat label transfer scores and DAR strength, we downsampled each experiment to 2,500, 2,000, 1,500, 1,000 and 500 total cells. Here, both Seurat label transfer scores and DAR strength were positively affected by higher cell counts (Fig. 3a and Extended Data Fig. 8a,c), but the total number of DARs recovered remained largely unaffected (Extended Data Fig. 8b). To mitigate count-dependent biases, we recalculated cell-type-specific DARs in eight technology-exclusive sets sampled from the initially merged 169,000 cells. Each technology-exclusive set contained the lowest number of cells available for each cell type across technologies: 555 B cells, 747 CD14^+^ monocytes, 1,008 naive T cells, 1,769 cytotoxic T cells, 83 DCs, 126 NK cells and 144 CD16^+^ monocytes (Extended Data Fig. 4a). In this cell count-balanced analysis, 10x methods recovered more DARs than ddSEQ and HyDrop across all cell types (Fig. 3b). s3-ATAC also recovered a large number of DARs but of strongly decreased strength compared to other techniques (Fig. 3a). In terms of DAR strength, 10x v1 and v2 performed best, with ddSEQ performing on par with the remaining 10x methods.

Additionally, we calculated cell-type-specific DARs on a merged dataset containing all eight aforementioned cell-type-balanced subsets. A comparison of the scATAC-seq signal at the strongest DAR (Fig. 3d) or across the top 2,000 strongest DARs per cell type (Supplementary Fig. 3) showed general agreement in signals between the 10x methods and Bio-Rad ddSEQ. Both s3-ATAC and HyDrop showed weaker signals around DARs. Specifically, the signal was more broadly distributed around the DAR periphery in s3-ATAC, whereas the absolute signal was weaker but more specific in HyDrop. This observation agreed with earlier quality metrics indicating higher fragment count and lower FRIP for s3-ATAC and the inverse for HyDrop. We then calculated the overlap between these consensus DARs and the DARs recovered by each of the eight cell-type-balanced sets. Both 10x and Bio-Rad ddSEQ largely agreed with the strongest consensus peaks, sharing between 85% and 97% of these regions (Fig. 3e and Extended Data Fig. 4b). s3-ATAC and HyDrop recovered 60% and 70%, respectively. However, ddSEQ recovered a significantly lower number of DARs from the consensus DAR set (40%; Fig. 3f). Across all eight individual ddSEQ samples, DARs were also positioned closer to TSSs than in all other techniques (Fig. 2j and Extended Data Fig. 4c). Combined with ddSEQ’s significantly higher TSS enrichment across all fragments, this suggests that ddSEQ recovers TSS-proximal DARs in agreement with 10x but lacks enrichment in TSS-distal fragments. In the merged set, we also calculated DARs between cell populations of the same cell type but originating from different techniques. Here, only s3-ATAC returned significantly enriched regions, finding a total of 193,913 regions that were more accessible in s3-ATAC than in the other techniques.

To evaluate the biological relevance of the DARs identified by each protocol, we performed a motif enrichment analysis on each cell type’s top 2,000 strongest DARs by using pyCisTarget^24–26^. Across all cell types, DARs from 10x methods scored highest in normalized enrichment scores of transcription factor motifs, with mean scores of higher than 10 across the top 50 most enriched motifs. ddSEQ, s3-ATAC and HyDrop scored significantly lower, with mean scores of 6–8. This stratification was conserved when examining cell-type-specific enriched motifs (Fig. 3g).

### Interpretation, integration and validation

**Fig. 4:**
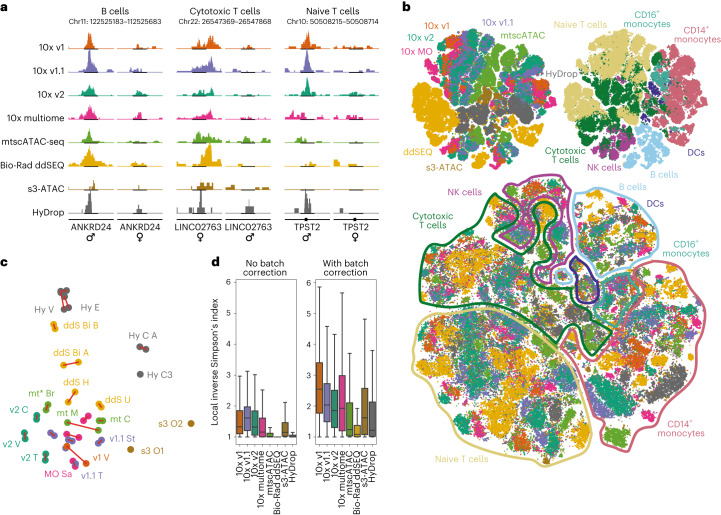
Overview of the merged dataset. **a**, Genome tracks of sex-specific DARs in B cells and cytotoxic and naive T cells showing coverage in male and female subpopulations. Coverage uses a common scale across techniques within each cell type and is cell count normalized (cell counts are equal for each sex within techniques); Chr, chromosome. **b**, Top, *t*-distributed stochastic neighbor embedding (t-SNE) projection of all 167,000 filtered cells across all 47 experiments colored by technology and cell type. Bottom, batch-corrected t-SNE using technology of origin as the batch variable. Data points were randomly shuffled before plotting. **c**, First two principal components of all experiments based on 15 basic quality metrics. Red links indicate same day replicate experiments. Caption denotes technique followed by center of sample origin (B for BioRad, Br for Broad, C for CNAG-CRG, E for EPFL, H for Harvard, M for MDC, O for OHSU, Sa for Sanger, St for Stanford, T for 10x Genomics, U for UCSF, and V for VIB), and finally an index (either A or B) to denote the replicate set if more than one set of replicates was performed at the same center. **d**, Local inverse Simpson index values before and after Harmony batch correction. Source data

**Extended Data Fig. 2:**
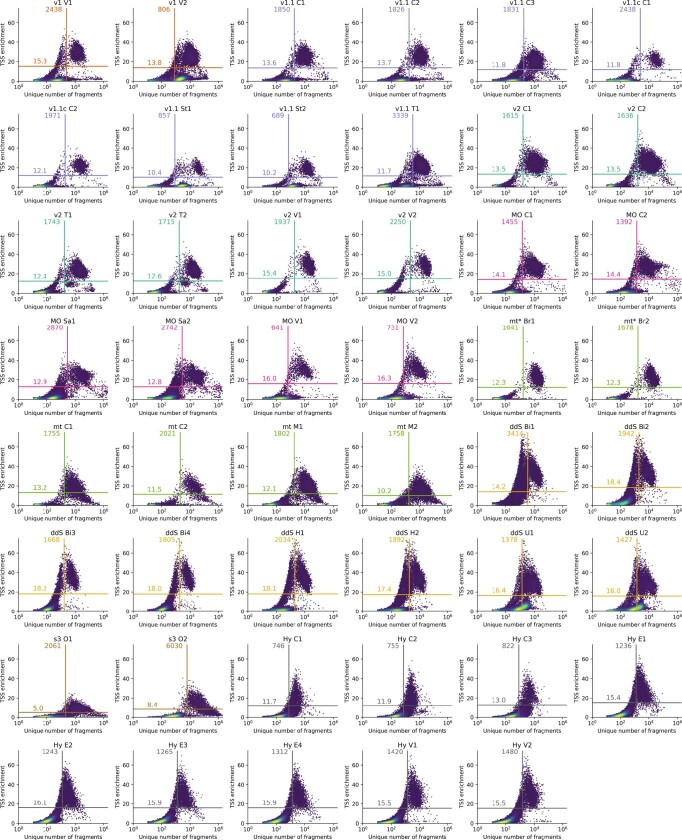
Individual TSS enrichment and Unique number of fragments distributions for each sample. Thresholds on both axes calculated using Otsu’s algorithm. Barcodes meeting both thresholds (upper right quadrant) are identified as cells in further analysis.

**Extended Data Fig. 3:**
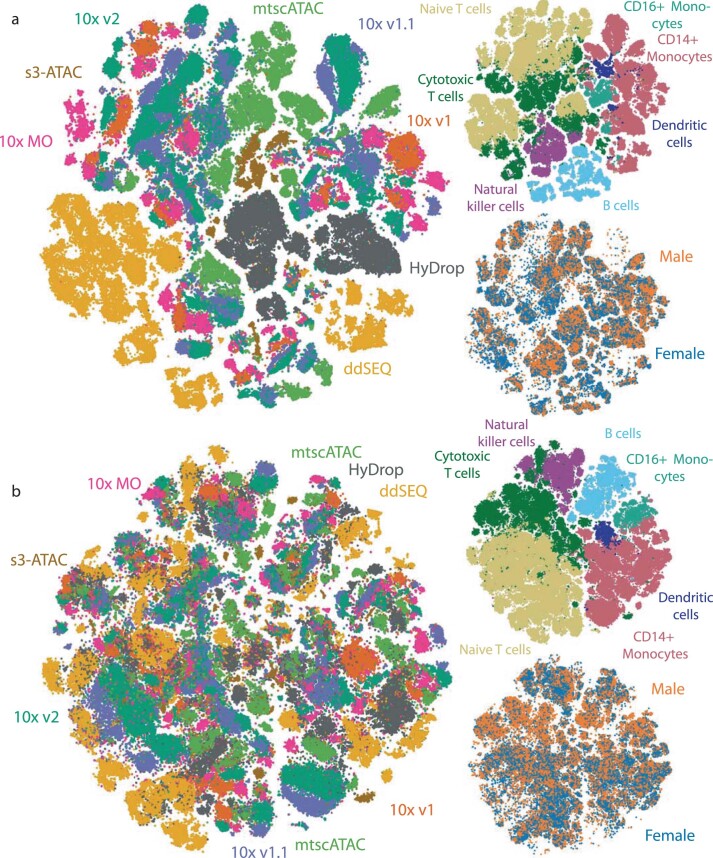
tSNE of full, non-downsampled data. Datapoints were randomly shuffled before plotting. **a**, Non-harmony-corrected tSNE of non-downsampled data, colored by technology of origin, cell type and donor identity. **b**, Harmony-corrected (by technology, considering 10x v1, 1.1, 2 as a single technology) tSNE of non-downsampled data, colored by technology of origin, cell type and donor identity.

Above, we calculated DARs between cell types within a sample. For biological discoveries, often different samples are compared to identify genomic regions with differential accessibility in certain conditions (for example, disease versus healthy and knockout versus control). To compare each method’s ability to detect variation between samples, where differences can be more subtle than between cell types, we focused on differences observed between male and female samples. For donor identification, we first applied Freemuxlet for genotype demultiplexing^20^ (Supplementary Fig. 2a–c) before assigning each sample’s sex through count numbers on the X and Y chromosomes (Extended Data Fig. 2b). Similar to the previously applied doublet calling, Freemuxlet’s donor assignment confidence was strongly correlated with the number of fragments (Fig. 3h). From a biological perspective, the ratio of naive to cytotoxic T cells was higher in the female sample than in male cells across all techniques, an effect dependent on the number of unique fragments recovered per cell (Fig. 3i). Sensitivity will thus directly impact studies that assume correct donor identification. We then used the aforementioned cell count-balanced strategy to calculate DARs between male and female samples for each cell type. Here, differences were markedly more subtle than between cell types, with median DAR enrichments of around 2-fold (Fig. 3k) as opposed to 5-fold in DCs or 50-fold in B cells (Fig. 3c). The strongest sexual dimorphism was observed in cytotoxic T cells, where sex-specific DARs were strongly enriched and highest in number (Figs. 3j,k and 4a). In agreement with these findings, sex differences in T cell abundance and chromatin accessibility have been previously reported in the context of naive/cytotoxic T cell counts and distinct response to extrinsic stimulation^27^. Similar to results obtained for cell-type-specific DARs, ddSEQ captured fewer and weaker DARs than the 10x methods, although the differences were less pronounced. Both s3-ATAC and HyDrop recovered even fewer and weaker or no sex-specific DARs (Fig. 3j,k).

Next, we tested each method’s ability to be integrated into joint datasets, a key requirement for decentralized multicenter projects, such as the Human Cell Atlas project. From the peak sets of the 47 individual downsampled samples, we derived a consensus set of 198,421 peaks and recounted all downsampled data in this common peak set to generate a complete merged dataset of 178,502 cells. After filtering for doublets, we performed dimensionality reduction on the remaining 169,227 cells using cisTopic (Fig. 4b). We then quantified each technology’s ability to cocluster with other techniques using the local inverse Simpson’s index, depicting the average number of technologies in a cell’s neighborhood (Fig. 4d)^28^. Before batch effect correction, cells clustered by technology, even across the different 10x variants. After data integration using Harmony, differences were partially remediated, although ddSEQ and HyDrop integrated worse than the other methods (Fig. 4b–d). Additionally, we performed a principal-component analysis on 15 key quality metrics (Fig. 4c). This dimension-reduced representation exhibited a radial axis of ‘quality’ centered around the bottom lefthand corner and showed that technical replicate experiments produced highly similar datasets.

In addition to the systematic benchmarking using PBMCs, we applied our universal data analysis strategy to publicly available adult mouse cortex scATAC-seq data for each of the eight technologies. Therefore, the raw sequencing data were aligned to the mouse reference genome, downsampled to equal cell numbers and clustered using cisTopic before generating consensus peak sets for each sample. We then calculated basic quality metrics, such as unique fragment numbers in peaks, and repeated these steps for several sequencing depth downsampling levels. In all metrics, 10x and ddSEQ performed markedly better than HyDrop and s3-ATAC (Extended Data Fig. 9a,b). However, in this tissue context, s3-ATAC recovered more unique fragments in peaks than HyDrop at all levels of downsampling. Surprisingly, considering previous results, 10x v1 recovered more unique fragments in peaks than 10x v2, the latter of which now performed on par with the best Bio-Rad ddSEQ samples. While our PBMC benchmark sought to systematically eliminate sample preparation effects by using cells in suspension as a reference sample, we cannot entirely disentangle the effect of sample (nuclei) preparation differences on technology performance in more complex tissues, such as the mouse cortex. Here, tissue excision and nuclei extraction require more complex procedures and expertise, which may be a more defining factor for data quality than the intrinsic sensitivity of each technology.

### Protocol economics

To compare costs per sample across the tested methods, we iteratively downsampled the fragments files for each sample and calculated the fraction of duplicate fragments per iteration. We then calculated the average sequencing depth at which a duplication rate of 50% was attained and quantified the number of fragments at this depth (including non-peak fragments; Extended Data Fig. 10). These findings provided an estimated total cost for a 5,000-cell experiment for each technique. In this regard, the costs per cell were fivefold and tenfold cheaper for HyDrop than for Bio-Rad ddSeq and 10x v2, respectively; 10x multiome was more expensive (1.5-fold) than the stand-alone assay 10x v2 but was markedly less sensitive and less efficient in sequencing. Thus, the matched gene expression information of the multiome assay results in a considerable additional cost, which has to be taken into account in designs for which scATAC-seq is the primary focus of a study. The cost per experiment for s3-ATAC ($800) ranged between HyDrop ($100) and Bio-Rad ddSEQ ($1,100), but the high library complexity resulted in high sequencing costs and the highest overall costs per cell. However, several variables can be tuned to reduce cost or to increase efficiency. For example, 10,000 cells (instead of 5,000 cells) can be loaded on the same microfluidic lane to double cost efficiency in the droplet-based methods (10x, Bio-Rad ddSEQ and HyDrop) or s3-ATAC samples can be sequenced at lower depths to reduce sequencing costs. All technologies can be sequenced at increased depths to improve sensitivity at the expense of cost efficiency.

## Discussion

Technology benchmarking studies with multicenter designs require thorough experimental planning to limit variability to the tested protocols, while keeping other factors constant. In this regard, we used PBMCs as a reference sample, which is ideal for multicenter benchmarking studies as cells can be aliquoted, stored, shipped and thawed without major losses in sample quality and composition. However, although such unified reference samples allowed us to include expert laboratories and companies around the world, we cannot exclude different performances of the methods in other tissues. Although the analysis of mouse brain datasets pointed to largely consistent method performance, a protocol could outperform other methods depending on the cell type and tissue context. Data analysis represents an additional variable in benchmarking studies, as protocol-specific pipelines harbor quality control and filtering steps that challenge comparative results if not synchronized. Therefore, we developed PUMATAC, a unified data preprocessing pipeline, which was used to process data types from all methods tested here. Importantly, its modular design allows the extension to future technologies and assay variants, making it a valuable software resource to benchmark the next generation of scATAC-seq methods.

Our reference sample resource and unified data processing pipeline allowed a systematic comparison of different scATAC-seq methods. Methods broadly agreed on cell-type identity and transcription factor activities but showed stark differences in sequencing library quality and tagmentation specificity to open chromatin sites. In general, HyDrop and s3-ATAC performed markedly lower in most quality control metrics. HyDrop captured significantly fewer fragments than 10x and Bio-Rad methods. s3-ATAC fragments were less likely to be enriched around TSSs, and high duplicate rates were observed in HyDrop, suggesting room for optimization in PBMC samples for these non-commercial technologies. Although the 10x protocols we tested apply similar chemistries, we see variable performance between variants and replicates. While the former points to the potential impact of buffer composition (for example, specific to mtscATAC and 10x multiome), the latter suggests differences due to sample handling. Nevertheless, despite the difference in number of unique fragments detected, 10x v1 and v2 scored equally across higher-level quality metrics, such as label transfer and motif enrichment scores, attesting to the high specificity and signal-to-noise ratio of the 10x v1 scATAC-seq kits. Bio-Rad ddSEQ samples returned weaker and fewer DARs, which in turn recovered weaker transcription factor motif signatures. Bio-Rad ddSEQ fragments were also less specific to TSS-distal accessible sites, which could also contribute to reduced integration capacity with other methods, an effect that could only partially be resolved using batch effect correction algorithms.

We found sequencing efficiency to be generally low for scATAC-seq experiments. Two strategies could mitigate this issue: optimized sample preparation and nuclei extraction protocols to minimize the amount of ambient chromatin in samples, potentially applying FACS for sample cleanup, and sequencing below library saturation to limit the number of duplicate reads. Generally, before embarking into large-scale production, the higher reagent costs of commercialized methods have to be considered in light of lower complexity and accuracy for non-commercial variants. The aforementioned factors plus a method’s accessibility and dataset integrability across studies should eventually drive the technology selection process. It is important to state that non-commercialized methods have not gone through a rigorous optimization process. Time and budget constraints of academic labs often limit excessive protocol optimization procedures. However, their open-source character and low reagent costs make them available for community-driven improvements and low-budget data generation effort, respectively. Therefore, we encourage researchers to continue striving for new creative solutions as the driving force for next-generation technologies.

In addition to evaluating different scATAC-seq methods, this work provides resources for the single-cell genomics community. Our PUMATAC pipeline is openly available to be used as an open-source alternative to commercial software and is flexible to analyze current and future data types. All code to reproduce all of our analyses and graphs is included with the pipeline. All benchmarking datasets can be downloaded in raw and processed formats for the testing and development of computational algorithms, for example, data integration tools. Finally, the here-derived set of consensus DARs across different technologies represents a high-confidence set of candidate enhancers and promoters underlying cell identity as a resource for further exploration.

## Methods

### Sample preparation

#### Human PBMC thawing and nuclei isolation

Cryopreserved human PBMCs from one male donor and one female donor were purchased from AllCells and distributed across institutes to generate the following samples: v1 V1, v1 V2, v1.1 C1–v1.1 C3, v1.1 St1, v1.1 St2, v1.1c C1, v1.1c C2, v2 V1, v2 V2, v2 C1, v2 C2, MO Sa1, MO Sa2, MO C1, MO C2, MO V1, MO V2, mt M1, mt M2, mt C1, mt C2, mt* Br1, mt* Br2, ddS H1, ddS H2, s3 O1, Hy E1–Hy E4, Hy V1, Hy V2 and Hy C1–Hy C3. For the remaining samples (v1.1 T1, v2 T1, v2 T2, ddS Bi1–ddS Bi4, ddS U1, ddS U2 and s3 O2), locally available cryopreserved PBMCs were used. In these short identifiers, the first part indicates the technology used, the second part indicates the first one or two letters from the center where the experiment was performed, and the number identifies each technical replicate.

Unless specified otherwise in technology-specific methods sections (for Bio-Rad ddSEQ, mtscATAC-seq, 10x v1.1 control runs and s3-ATAC), cryopreserved PBMCs were thawed according to the 10x Genomics demonstrated protocol CG00039 (‘Fresh Frozen Human Peripheral Blood Mononuclear Cells for Single Cell RNA Sequencing’). Briefly, 1 ml of frozen cells was rapidly thawed in a water bath at 37 °C and transferred to a 50-ml tube using a 1,000-µl wide-bore tip. Next, 1 ml of medium prewarmed to 37 °C and supplemented with 10% fetal bovine serum (FBS; Thermo Fisher Scientific) was added dropwise with gentle swirling of the sample. After 1 min of incubation at room temperature, 2, 4, 8 and 16 ml of medium with 10% FBS were added dropwise with 1 min of incubation at room temperature in between. The cell suspension was then centrifuged at 300*g* for 5 min at room temperature. The pellet was resuspended in 10 ml of medium supplemented with 10% FBS, and cells were counted. Unless specified otherwise in the technology-specific methods sections, the isolation of nuclei was performed according to the 10x Genomics demonstrated protocol ‘Nuclei Isolation for Single Cell ATAC Sequencing’. Briefly, 1 million cells from the cell mix were transferred to a 1.5-ml microcentrifuge tube and centrifuged at 500*g* for 5 min at 4 °C. The supernatant was removed without disrupting the cell pellet, and 100 µl of chilled lysis buffer (10 mM Tris-HCl (pH 7.4), 10 mM NaCl, 3 mM MgCl_2_, 0.1% Tween 20, 0.1% NP-40 substitute, 0.01% digitonin and 1% bovine serum albumin (BSA)) was added and mixed by pipetting ten times. Samples were then incubated on ice for 3 min. Following lysis, 1 ml of chilled wash buffer (10 mM Tris-HCl (pH 7.4), 10 mM NaCl, 3 mM MgCl_2_, 0.1% Tween 20 and 1% BSA) was added and mixed by pipetting. Nuclei were centrifuged at 500*g* for 5 min at 4 °C, and the supernatant was removed without disrupting the nuclei pellet. Based on the starting number of cells and assuming a 50% loss during the procedure, nuclei were resuspended into the appropriate volume of chilled diluted Nuclei Buffer (10x Genomics) to achieve a concentration of 925–2,300 nuclei per µl, suitable for a target recovery of 3,000 nuclei. This combination of PBMC thawing and nuclei isolation was used for all 10x samples (except mtscATAC-seq protocols, v1.1 control runs and v1.1 St1 and v1.1 St2 samples) and all HyDrop samples, but not for s3-ATAC and Bio-Rad ddSEQ samples. The method of cell counting was performed differently depending on the center of origin. For all samples generated in VIB, cells and nuclei were counted using a LUNA automated cell counter (Logos Biosystems). For Stanford and Sanger samples, cells and nuclei were counted manually using a hemocytometer. For all Bio-Rad ddSEQ and s3-ATAC and all CNAG samples, cells and nuclei were counted using a TC20 cell counter (Bio-Rad). For Broad samples and samples generated by the company 10x Genomics, cells and nuclei were counted using a Countess II or III FL automated cell counter (Thermo Fisher).

#### 10x ATAC v1 (short identifiers v1 V1 and v1 V2)

PBMCs were thawed, and nuclei were isolated as described above. Two technical replicates were generated on the same day starting from the same freshly thawed nuclei suspension. scATAC-seq libraries were prepared according to the Chromium Single Cell ATAC reagent kits v1.0 user guide (10x Genomics, CG000001 Rev D). Briefly, the transposition reaction was prepared by mixing the desired number of nuclei with ATAC Buffer (10x Genomics) and ATAC Enzyme (10x Genomics) and incubated for 60 min at 37 °C; 4,590 nuclei were loaded with the goal of recovering 3,000 nuclei. Nuclei were partitioned into Gel Bead-in-Emulsions (GEMs) by using the Chromium Controller (Chip E). DNA linear amplification was then performed by incubating the GEMs under the following thermal cycling conditions: 72 °C for 5 min, 98 °C for 30 s and 12 cycles of 98 °C for 10 s, 59 °C for 30 s and 72 °C for 1 min. GEMs were broken using Recovery Agent (10x Genomics), and the resulting DNA was purified by sequential Dynabeads and SPRIselect reagent beads cleanups. Libraries were indexed by PCR using a Single Index kit (Plate N) and incubating under the following thermal cycling conditions: 98 °C for 45 s and ten cycles of 98 °C for 20 s, 67 °C for 30 s and 72 °C for 20 s with a final extension of 72 °C for 1 min. Sequencing libraries were subjected to a final bead cleanup with SPRIselect reagent.

Samples v1 V1 and v1 V2 were sequenced on a NovaSeq 6000 using a NovaSeq SP kit (100 cycles; 20028401, Illumina), and sequencing was performed using the following read protocol: 50 cycles (read 1), 8 cycles (i7 index read), 16 cycles (i5 index read) and 49 cycles (read 2).

#### 10x ATAC v1.1 (short identifiers v1.1 C1–v1.1 C3, v1.1 T1, v1.1 St1 and v1.1 St2)

PBMCs were thawed, and nuclei were isolated as described above for samples v1.1 C1–v1.1 C3 and v1.1 T1. For samples v1.1 St1 and v1.1 St2, a different thawing/isolation protocol was used. Here, each cryopreserved PBMC sample was thawed in 50 ml of thaw medium (IMDM, 10% FBS and 200 Kunitz U ml^–1^ DNase) preheated to 37 °C and incubated for 15 min at 37 °C. DNase was ordered from Worthington Biochem (LS002007) and resuspended in HBSS at 20,000 U ml^–1^ (100× stock). Cells were pelleted at 300*g* (1,200 r.p.m.), resuspended in 5 ml of thaw medium and layered over 5 ml of Ficoll in a 15-ml conical tube. Cells were then spun at 500*g* (1,500 r.p.m.) with no brake for 30 min at room temperature in a swinging-bucket centrifuge. 2 mL of the mononuclear cell layer was collected and diluted with 10 ml of room temperature PBS. Cells were put on ice and maintained at 4 °C until use.

Technical replicates were generated on the same day starting from the same freshly thawed nuclei suspension. scATAC-seq libraries were prepared according to the Chromium Single Cell ATAC reagent kit v1.1 user guide (10x Genomics, CG000209 Rev D). Briefly, the transposition reaction was prepared by mixing the desired number of nuclei with ATAC Buffer (10x Genomics) and ATAC Enzyme (10x Genomics) and was then incubated for 60 min at 37 °C; 4,590 nuclei were loaded with the goal of recovering 3,000 nuclei. For sample ‘10x v1.1 V2’, 9,180 nuclei were loaded instead of 4,590 due to a counting error. Nuclei were partitioned into GEMs by using a Chromium Controller with Chip H. DNA linear amplification was then performed by incubating the GEMs under the following thermal cycling conditions: 72 °C for 5 min, 98 °C for 30 s and 12 cycles of 98 °C for 10 s, 59 °C for 30 s and 72 °C for 1 min. GEMs were broken using Recovery Agent (10x Genomics), and the resulting DNA was purified by sequential Dynabeads and SPRIselect reagent beads cleanups. Libraries were indexed by PCR using a Single Index kit N set A (10x Genomics, PN-1000212) and incubated under the following thermal cycling conditions: 98 °C for 45 s and ten cycles of 98 °C for 20 s, 67 °C for 30 s and 72 °C for 20 s with a final extension of 72 °C for 1 min. Sequencing libraries were subjected to a final bead cleanup with SPRIselect reagent.

Samples v1.1 St1 and v1.1 St2 were sequenced on an Illumina NextSeq 500 machine using a high-output flow cell with 34 bp paired-end reads. Samples v1.1 C1–v1.1 C3 and v1.1 T1 were sequenced on an Illumina NovaSeq 6000 with the following sequencing conditions: 50 bp (read 1), 8 bp (i7 index), 16 bp (i5 index) and 49 bp (read 2).

#### 10x ATAC v2 (short identifiers v2 V1, v2 V2, v2 T1, v2 T2, v2 C1 and v2 C2)

PBMCs were thawed, and nuclei were isolated as described above. Technical replicates were generated on the same day starting from the same freshly thawed nuclei suspension. scATAC-seq libraries were prepared according to the Chromium Single Cell ATAC reagent kits v2 user guide (10x Genomics, CG000496 Rev B). Briefly, the transposition reaction was prepared by mixing the desired number of nuclei with ATAC Buffer (10x Genomics) and ATAC Enzyme (10x Genomics) and was then incubated for 30 min at 37 °C; 4,590 nuclei were loaded with a goal of recovering 3,000 nuclei. Nuclei were partitioned into GEMs by using a Chromium Controller with Chip H. Sample v2 T1 was the only sample for which Chromium X was used. DNA linear amplification was then performed by incubating the GEMs under the following thermal cycling conditions: 72 °C for 5 min, 98 °C for 30 s and 12 cycles of 98 °C for 10 s, 59 °C for 30 s and 72 °C for 1 min. GEMs were broken using Recovery Agent (10x Genomics), and the resulting DNA was purified by sequential Dynabeads and SPRIselect reagent beads cleanups. Libraries were indexed by PCR using a Single Index kit N set A and incubated under the following thermal cycling conditions: 98 °C for 45 s and eight cycles of 98 °C for 20 s, 67 °C for 30 s and 72 °C for 20 s with a final extension of 72 °C for 1 min. Sequencing libraries were subjected to a final bead cleanup with SPRIselect reagent.

Samples v2 v1 and v2 v2 were sequenced on an Illumina NextSeq 2000 under the following sequencing conditions: 50 bp (read 1), 8 bp (i7 index), 16 bp (i5 index) and 50 bp (read 2). Samples v2 C1, v2 C2, v2 T1 and v2 T2 were sequenced on an Illumina NovaSeq 6000 under the following sequencing conditions: 50 bp (read 1), 8 bp (i7 index), 16 bp (i5 index) and 49 bp (read 2).

#### 10x multiome (short identifiers MO Sa1, MO Sa2, MO C1, MO C2, MO V1 and MO V2)

PBMCs were thawed as described above. The isolation of nuclei was slightly different, including the use of RNase inhibitors to ensure RNA quality. Briefly, two pools of cells (technical replicates) were generated from the two donors by mixing 500,000 cells per donor, totaling 1 million cells per pool. Cells were pelleted for 5 min at 300*g* and 4 °C and were washed twice in 1 ml of wash buffer (10 mM Tris-HCl (pH 7.4), 10 mM NaCl, 3 mM MgCl_2_, 1% BSA, 0.1% Tween 20, 1 mM DTT and 1 U µl^–1^ RNase inhibitor). After the second wash and final centrifugation, cells were resuspended in 0.1 ml of chilled lysis buffer (10 mM Tris-HCl (pH 7.4), 10 mM NaCl, 3 mM MgCl_2_, 0.1% Tween 20, 0.1% NP-40, 0.01% digitonin, 1% BSA and 1 mM DTT) and incubated for 3 min on ice. Nuclei were washed three times in 1 ml of wash buffer (10 mM Tris-HCl (pH 7.4), 10 mM NaCl, 3 mM MgCl_2_, 1% BSA, 0.1% Tween 20, 1 mM DTT and 1 U µl^–1^ RNase inhibitor) by centrifuging at 500*g* for 5 min.

After the last centrifugation, cells were resuspended in chilled Nuclei Buffer (1× Nuclei Buffer, 1 mM DTT and 1 U µl^–1^ RNase inhibitor) calculated and loaded according to the Chromium Next GEM Single Cell Multiome ATAC + GEX user guide (protocol CG000338 Rev A); 4,590 nuclei were loaded with the goal of recovering 3,000 nuclei. For loading onto 10x chips, we sought to recover 3,000 nuclei. Following the isolation of nuclei and transposition, GEMs were generated using GEM Chip J. GEM cleanup and preamplification PCR were performed as per the user guide. For the ATAC-seq library, eight cycles of PCR were run, while seven cycles of PCR were performed for cDNA amplification. Of the amplified cDNA, 25% of the material was used for gene expression library construction with 15 cycles of PCR for both technical replicates.

For samples MO Sa1, MO Sa2, MO C1 and MO C2, ATAC libraries were sequenced on an Illumina NovaSeq 6000 using the following read protocol: 50 cycles (read 1), 8 cycles (i7 index read), 24 cycles (i5 index read) and 49 cycles (read 2). ATAC libraries from MO V1 and MO V2 were sequenced according to the same parameters but on a NextSeq 2000. For samples MO Sa1, MO Sa2, MO C1 and MO C2, RNA libraries were sequenced on an Illumina NovaSeq 6000 using the following read protocol: 28 cycles (read 1), 10 cycles (i7 index read), 10 cycles (i5 index read) and 90 cycles (read 2). RNA libraries from MO V1 and MO V2 were sequenced according to the same parameters but on a NextSeq 2000.

#### 10x mtscATAC (short identifiers mt M1, mt M2, mt C1, mt C2, mt* Br1 and mt* Br2)

Cryopreserved PBMCs were thawed as described above. For samples mt* Br1 and mt* Br2, cells were also washed, and 250,000 live cells were sorted using SytoxBlue at a 1:1,000 dilution as a live/dead cell stain. Samples mt M1, mt M2, mt C1 and mt C2 were not sorted. Cells from each donor were subsequently pooled at a 1:1 ratio, and, after washing, cells were fixed in 1% formaldehyde (Thermo Fisher, 28906) in PBS for 10 min at room temperature, quenched with glycine solution to a final concentration of 0.125 M and washed twice in PBS via centrifugation at 400*g* for 5 min at 4 °C. Cells were subsequently treated with lysis buffer (10 mM Tris-HCl (pH 7.4), 10 mM NaCl, 3 mM MgCl_2_, 0.1% NP-40 and 1% BSA) for 3 min on ice, followed by the addition of 1 ml of chilled wash buffer and inversion (10 mM Tris-HCl (pH 7.4), 10 mM NaCl, 3 mM MgCl_2_ and 1% BSA) before centrifugation at 500*g* for 5 min at 4 °C. The supernatant was discarded, and cells were diluted in 1× diluted Nuclei Buffer before counting using trypan blue and a Countess II FL automated cell counter. Subsequently, mtscATAC-seq libraries were generated using the Chromium Next GEM Single Cell ATAC Library & Gel Bead kit (v1.1, 1000175) according to the manufacturer’s instructions (CG000209); 4,590 nuclei were loaded with the goal of recovering 3,000 nuclei. Briefly, following tagmentation, cells were loaded onto a Chromium Controller Single Cell instrument to generate single-cell GEMs, followed by linear PCR, as described in the protocol using a C1000 Touch thermal cycler with the 96-Deep Well Reaction Module (Bio-Rad). After breaking the GEMs, barcoded tagmented DNA was purified and further amplified to enable sample indexing (11 cycles of PCR) and enrichment of mtscATAC-seq libraries. The final libraries were quantified using a Qubit double-stranded DNA high-sensitivity assay kit (Invitrogen) and a high-sensitivity DNA chip run on a Bioanalyzer 2100 system (Agilent).

Samples mt C1 and mt C2 were sequenced on an Illumina NovaSeq 6000 under the following sequencing conditions: 50 bp (read 1), 8 bp (i7 index), 16 bp (i5 index) and 49 bp (read 2). Samples mt M1 and mt M2 were sequenced on an Illumina NovaSeq 6000 under the following sequencing conditions: 150 bp (read 1), 8 bp (i7 index), 16 bp (i5 index) and 150 bp (read 2). Samples mt* Br1 and mt* Br2 were sequenced on an Illumina Nextseq 550 with paired-end reads (2 × 72 cycles), 8 cycles for index 1 and 16 cycles for index 2.

#### Bio-Rad SureCell ATAC (short identifiers ddS Bi1–ddS Bi4, ddS H1, ddS H2, ddS U1 and ddS U2)

Cryopreserved PBMCs were quickly thawed in a water bath at 37 °C, rinsed with culture medium (RPMI supplemented with 15% FBS) and treated with 0.2 U μl^−1^ DNase I (Thermo Fisher Scientific) in 5 ml of culture medium at 37 °C for 30 min. After DNase I treatment, cells were washed once with medium and twice with ice-cold 1× PBS supplemented with 0.1% BSA. Cells were then filtered with a 35-μm cell strainer (Corning), and cell viability and concentration were measured with trypan blue on a TC20 automated cell counter (Bio-Rad).

For a detailed description of tagmentation protocols and buffer formulations, refer to the SureCell ATAC-Seq Library Prep kit user guide (17004620, Bio-Rad). Collected cells and tagmentation buffers were chilled on ice. Lysis was performed simultaneously with tagmentation. After washing, equal numbers of cells from each donor were mixed with Whole-Cell Tagmentation Mix containing 0.1% Tween 20, 0.01% digitonin and 1× PBS supplemented with 0.1% BSA, ATAC Tagmentation Buffer and ATAC Tagmentation Enzyme (ATAC Tagmentation Buffer and ATAC Tagmentation Enzyme are both included in the SureCell ATAC-Seq Library Prep kit (17004620, Bio-Rad)). The mix was split into two technical replicates, and cells were then mixed and agitated on a ThermoMixer (5382000023, Eppendorf) for 30 min at 37 °C. Tagmented cells were kept on ice before encapsulation.

Tagmented cells were loaded onto a ddSEQ Single-Cell Isolator (12004336, Bio-Rad). For samples ddS H1, ddS H2, ddS U1 and ddS U2, 5,000 nuclei were loaded with the goal of recovering 3,000 nuclei. scATAC-seq libraries were prepared using a SureCell ATAC-Seq Library Prep kit (17004620, Bio-Rad) and SureCell ddSEQ Index kit (12009360, Bio-Rad). Bead barcoding and sample indexing were performed in a C1000 Touch thermal cycler with a 96-Deep Well Reaction Module (1851197, Bio-Rad). The following PCR conditions were used: 37 °C for 30 min; 85 °C for 10 min; 72 °C for 5 min; 98 °C for 30 s; eight cycles of 98 °C for 10 s, 55 °C for 30 s and 72 °C for 60 s and a single 72 °C extension for 5 min to finish. Emulsions were broken, and products were cleaned up using Ampure XP beads (A63880, Beckman Coulter). Barcoded amplicons were further amplified using a C1000 Touch thermal cycler with a 96-Deep Well Reaction Module. The following PCR conditions were used: 98 °C for 30 s and seven cycles of 98 °C for 10 s, 55 °C for 30 s and 72 °C for 60 s and a single 72 °C extension for 5 min to finish. PCR products were purified using Ampure XP beads and quantified on an Agilent Bioanalyzer (G2939BA, Agilent) using a high-sensitivity DNA kit (5067-4626, Agilent).

For samples ddS H1, ddS H2, ddS Bi3, ddS Bi4, ddS U1 and ddS U2, libraries were sequenced on a NextSeq 550 (SY-415-1002, Illumina) using a NextSeq High-Output kit (150 cycles; 20024907, Illumina) and the following read protocol: 118 cycles (read 1), 8 cycles (i7 index) and 40 cycles (read 2). For ddS Bi1 and ddS Bi2, samples were sequenced on a NovaSeq according to the same protocol. A custom sequencing primer was required for read 1 (16005986, Bio-Rad; included in the kit).

#### HyDrop ATAC (short identifiers Hy E1–Hy E4, Hy V1, Hy V2 and Hy C1–Hy C3)

PBMCs were thawed, and nuclei were isolated as described above. HyDrop was performed as previously described^12^ but with an updated barcoded hydrogel bead design and minor improvements in nuclei handling. Briefly, barcoded hydrogel beads were produced as described previously but using 384 × 384 combinations of primers instead of the original method using 96 × 96 × 96 combinations, resulting in a barcode sequence of 30 bp instead of 50 bp. One million PBMCs were counted, pelleted and resuspended in 200 μl of ATAC Lysis Buffer (1% BSA, 10 mM Tris-HCl (pH 7.5), 10 mM NaCl, 0.1% Tween 20, 0.1% NP-40, 3 mM MgCl_2_, 70 μM Pitstop in DMSO and 0.01% digitonin) for 5 min on ice. One milliliter of ATAC Nuclei Wash Buffer (1% BSA, 10 mM Tris-HCl (pH 7.5), 0.1% Tween 20, 10 mM NaCl and 3 mM MgCl_2_) was added, and nuclei were pelleted at 500*g* at 4 °C for 5 min. The resulting pellet was resuspended in 100 μl of ice-cold PBS and filtered with a 40-μm strainer (Flowmi); 25,000 PBMC nuclei were resuspended in 25 μl of ATAC Reaction Mix (10% dimethylformamide, 10% Tris-HCl (pH 7.4), 5 mM MgCl_2_, 5 ng μl^–1^ Tn5, 70 μM Pitstop in DMSO, 0.1% Tween 20 and 0.01% digitonin) and incubated at 37 °C for 1 h without shaking. To recover a target of 3,000 nuclei, 5,625 tagmented nuclei were added to 48 μl of PCR mix (1.3× Phusion HF buffer, 15% OptiPrep, 1.3 mM dNTPs, 39 mM DTT, 0.065 U μl^–1^ Phusion HF polymerase, 0.065 U μl^–1^ Deep Vent polymerase and 0.013 U μl^–1^ ET SSB). PCR mix was coencapsulated with 35 μl of freshly thawed HyDrop ATAC beads in hydrofluoroether 7500 Novac oil with EA-008 surfactant (RAN Biotech) on an Onyx microfluidics platform (Droplet Genomics). The resulting emulsion was collected in aliquots of 25 μl in total volume and thermocycled according to the linear amplification program (72 °C for 15 min; 98 °C for 3 min; 12 amplification cycles of 98 °C for 10 s, 63 °C for 30 s and 72 °C for 1 min and a final hold at 4 °C). One hundred and twenty-five microliters of Recovery Agent (20% perfluorooctanol in hydrofluoroether 7500), 55 μl of guanidinium thiocyanate buffer (5 M guanidinium thiocyanate, 25 mM EDTA and 50 mM Tris-HCl (pH 7.4)) and 5 μl of 1 M DTT were added to each separate aliquot of 50 μl of thermocycled emulsion and incubated on ice for 5 min. Five microliters of Dynabeads was added to the aqueous phase and incubated for 10 min. Dynabeads were pelleted on a neodymium magnet and washed twice with 80% ethanol. Elution was performed in 50 μl of elution buffer (10 mM Tris-HCl, pH 8.5) supplemented with 10 mM DTT and 0.1% Tween 20. A 1× Ampure bead purification was performed according to manufacturer’s recommendations. Elution was performed in 30 μl of elution buffer supplemented with 10 mM DTT. Eluted library was further amplified in 100 μl of PCR mix (1× KAPA HiFi, 1 μM index i7 primer and 1 μM index i5 primer). The final library was purified in a 0.4–1.2× double-sided Ampure purification, eluted in 25 μl of elution buffer supplemented with 10 mM DTT and quality controlled on an Agilent Bioanalyzer high-sensitivity chip (Agilent Technologies).

Samples Hy V1, Hy V2 and Hy E1–Hy E4 were loaded at 750 pM on a NextSeq 2000 using a NextSeq 2000 P2 kit (100 cycles; 20046811, Illumina), and sequencing was performed using the following read protocol: 49 cycles (read 1), 10 cycles (i7 index read), 31 cycles (i5 index read) and 48 cycles (read 2). Samples Hy C1–Hy C3 were sequenced on a NovaSeq 6000 using the same parameters.

#### s3-ATAC (short identifiers s3 O1 and s3 O2)

Samples s3 O1 and s3 O2 were generated on different days according to the following protocol. Only sample s3 O1 was performed on the reference PBMC sample of two donors. The PBMC pellet was thawed and suspended in NIB-HEPES (pH 7.2; 10 mM HEPES-KOH (BP310-500 (Fisher Scientific) and 1050121000 (Sigma-Aldrich), respectively), 10 mM NaCl, 3 mM MgCl_2_ (Fisher Scientific, AC223210010), 0.1% (vol/vol) IGEPAL CA-630 (Sigma-Aldrich, I3021) and 0.1% (vol/vol) Tween (Sigma-Aldrich, P-7949)) before Dounce homogenization. s3-ATAC was then performed as described previously^13^. Two plates were prepared for a total of 2,880 nuclei per sample. Briefly, nuclei were flow sorted via a Sony SH800 to remove debris and attain an accurate count per well before PCR in 1× TD buffer. Immediately following sorting completion, the plate was sealed and centrifuged for 5 min at 500*g* and 4 °C to ensure that nuclei were within the buffer. Nucleosomes and remaining transposases were then denatured with the addition of 1 µl of 0.1% SDS (roughly 0.01% final concentration) per well. Then, 4 µl of NPM (Nextera XT kit, Illumina) per well was subsequently added to perform gap-fill on tagmented genomic DNA, with an incubation at 72 °C for 10 min. Next, 1.5 µl of 1 µM A14-LNA-ME oligonucleotides was added to supply the template for adapter switching. The polymerase-based adapter switching was then performed under the following conditions: initial denaturation at 98 °C for 30 s and ten cycles of 98 °C for 10 s, 59 °C for 20 s and 72 °C for 10 s. The plate was then held at 10 °C. After adapter switching, 1% (vol/vol) Triton X-100 in ultrapure water (Sigma, 93426) was added to quench persisting SDS. The following was then combined per well for PCR: 16.5 µl of sample, 2.5 µl of indexed i7 primer at 10 µM, 2.5 µl of indexed i5 primer at 10 µM, 3 µl of ultrapure water, 25 µl of NEBNext Q5U 2× master mix (New England Biolabs, M0597S) and 0.5 µl of 100× SYBR Green I (Thermo Scientific, S7563) for a total of 50 µl of reaction per well. Real-time PCR was performed on a Bio-Rad CFX under the following conditions measuring SYBR fluorescence every cycle: 98 °C for 30 s and 16–18 cycles of 98 °C for 10 s, 55 °C for 20 s and 72 °C for 30 s, fluorescent reading and 72 °C for 10 s. After fluorescence passed an exponential growth and began to inflect, the samples were held at 72 °C for another 30 s and stored at 4 °C. Amplified libraries were then cleaned by pooling 25 µl per well into a 15-ml conical tube and cleaning via a QIAquick PCR purification column following the manufacturer’s protocol (Qiagen, 28106). The pooled sample was eluted in 50 µl of 10 mM Tris-HCl (pH 8.0). Library molecules then went through size selection via SPRI selection beads (Mag-Bind TotalPure NGS Omega Biotek, M1378-01). Next, 50 µl of vortexed and fully suspended room temperature SPRI beads was combined with the 50-µl library (one cleanup) and incubated at room temperature for 5 min. The reaction was then placed on a magnetic rack, and, once cleared, the supernatant was removed. The remaining pellet was rinsed twice with 100 µl of fresh 80% ethanol. After the ethanol was pipetted out, the tube was spun down and placed back on the magnetic rack to remove any lingering ethanol. Next, 31 µl of 10 mM Tris-HCl (pH 8.0) was used to resuspend the beads off the magnetic rack, followed by an incubation for 5 min at room temperature. The tube was again placed on the magnetic rack, and, once cleared, the full volume of supernatant was moved to a clean tube. DNA was then quantified by Qubit double-stranded DNA high-sensitivity assay following the manufacturer’s instructions (Thermo Fisher, Q32851). Libraries were diluted to 2 ng µl^−1^ and run on an Agilent Tapestation 4150 D5000 tape (Agilent, 5067-5592). Library molecule concentration within the range of 100 to 1,000 bp was then used for final library dilution of 1 nM.

Samples s3 O1 and s3 O2 were sequenced on a NovaSeq S2 flow cell following the manufacturer’s recommendations (Illumina, 20028315) as paired-end libraries with 10 cycle index reads and 85 cycles (O1) or 90 cycles (O2) for reads 1 and 2.

#### 10x v1.1 control runs (short identifiers v1.1c C1 and v1.1c C2)

Two additional control runs were performed on the same day as v1.1 C3. Control run v1.1c C1 was performed using the standard 10x nuclei extraction lysis buffer with the omission of NP-40 to simulate the whole-cell protocol used in Bio-Rad ddSEQ experiments. Control run v1.1c C2 was performed using the Dounce homogenization protocol as described in the s3-ATAC experiments but without FACS. Starting from permeabilized cells or Dounce-extracted nuclei, both control runs were performed exactly according to the standard 10x v1.1 protocol simultaneously with v1.1 C3.

Samples v1.1c C1 and v1.1c C2 were sequenced on a NovaSeq 6000 with 50 cycles for read 1, 49 cycles for read 2, 8 cycles for index 1 and 16 cycles for index 2.

#### 10x scRNA-seq

Cryopreserved PBMCs were thawed as described above, and equal numbers of cells from each donor were mixed. The cell mix was partitioned into GEMs by using the Chromium Controller system (10x Genomics), with a target recovery of 5,000 total cells. We generated three technical replicates by loading three channels of Chip G with the same cell mix. cDNA sequencing libraries were prepared using the Next GEM Single Cell 3′ reagent kit v3.1 (10x Genomics, PN-1000268), following the manufacturer’s instructions. Briefly, after GEM-RT cleanup, cDNA was amplified during 12 cycles, and cDNA quality control and quantification were performed on an Agilent Bioanalyzer high-sensitivity chip (Agilent Technologies). cDNA libraries were indexed by PCR using the PN-220103 Chromium i7 Sample Index Plate. Size distribution and concentration of 3′ cDNA libraries were verified on an Agilent Bioanalyzer high-sensitivity chip (Agilent Technologies).

Sequencing of cDNA libraries was performed on an Illumina NovaSeq 6000 using the following sequencing conditions to obtain approximately 40,000 reads per cell: 28 bp (read 1), 8 bp (i7 index), 0 bp (i5 index) and 89 bp (read 2).

### Data preprocessing

#### Unified scATAC-seq data analysis pipeline (PUMATAC)

We developed PUMATAC, a unified Nextflow v21.04.3 (ref. ^29^) pipeline, to align samples from multiple technologies to the reference genome and write fragments files from these reference genome alignments (https://github.com/aertslab/PUMATAC). The steps implemented in PUMATAC are described briefly in the text below and in detail with examples at https://github.com/aertslab/scATAC-seq_benchmark. All code necessary to reproduce our analyses and graphics is present in notebooks in this repository.

#### Barcode correction and FASTQ processing (singlecelltoolkit in PUMATAC)

Each barcode was compared to the whitelist barcodes and kept (with bam tag ‘CB’) if it was a perfect match or if changing any of the bases by 1 bp resulted in a match (Hamming distance of 1). Barcodes that were unable to be corrected were retained with the ‘CR’ bam tag. The barcode tag information, including the original barcode quality scores (‘CY’), was added to the comments field in each of the two paired-end FASTQ files. Adapter trimming was then performed using TrimGalore (version 0.6.6)^30^ with the ‘–paired’ option, which in turn runs Cutadapt^31^.

#### Reference genome alignment and fragments writing (bwa-mem in PUMATAC)

We first aligned full sequencing datasets of all samples to the GRCh38 or mm10 reference genome using PUMATAC. We then filtered cells (described later) and downsampled all sequencing data to a common sequencing depth of 40,796 reads per cell and realigned these downsampled FASTQ files. In PUMATAC, alignment was performed using bwa-mem2 (v2.2.1)^32^ with the ‘mem’ method and default mapping parameters. The ‘-C’ option was used to copy the barcode tag information from the FASTQ file to the resulting bam file. Read group information was taken from the FASTQ name field in the first line of each input file and added with the ‘-R’ option in bwa-mem2. The ‘fixmate’ tool from SAMtools (version 1.12)^33^ was used to add mate coordinates and insert sizes to the file. Reads were aligned to the GRCh38 reference for the PBMC samples and to mm10 for mouse public data. From the resulting aligned reads in .bam format, fragments were written in the bed-like fragments.tsv.gz format using a combination of SAMtools^34^ and AWK, according to the base pair shift rules described in the CellRanger manual (https://support.10xgenomics.com/single-cell-atac/software/pipelines/latest/output/fragments).

#### Barcode multiplet detection (barcard in PUMATAC)

For each sample, we detected barcode multiplets using barcard, our own reimplementation of bap (https://github.com/caleblareau/bap). Similar to bap, barcard subsets fragments files to barcodes associated with at least 1,000 unique fragments. For each remaining barcode, the number of unique fragments that share their beginning and end coordinates between the two barcodes divided by the total number of barcodes found in both barcodes combined is calculated with every other barcode. The Jaccard indices for these barcode pairs are then ranked and thresholded using Otsu’s algorithm to identify barcode multiplets. Following the identification of barcode multiplets in each sample, a new tag (‘DB’) was added to the bam file to represent droplet barcodes. This tag contained either the original corrected barcode from the CB tag (in the case of singlets) or an underscore-separated concatenation of each corrected barcode that forms the multiplet. This step, and others, was parallelized using GNU Parallel^35^. Similarly, fragments.tsv.gz files were rewritten to merge detected barcode multiplets. While this step is only necessary for Bio-Rad ddSEQ samples, we detected and merged multiplets in all samples.

#### PUMATAC validation using CellRanger

We realigned all 10x v1, v1.1, v2 and multiome data using CellRanger-arc. We then subset fragments files generated by CellRanger and PUMATAC on barcodes identified as cell barcodes. For each pair of barcodes, we then calculated the number of unique fragments that were attributed to that barcode by both CellRanger and PUMATAC based on beginning and ending coordinates. The Jaccard index was calculated based on this number.

#### Downstream analyses

Starting from the fragments files generated by PUMATAC and merged using barcard, we then performed further analyses such as clustering, cell-type annotation, differential region calling and transcription factor motif analysis using a combination of bioinformatics packages.

#### Single-cell-level quality control and barcode filtering (pycisTopic)

We used the Python implementation of cisTopic^16^ (pycisTopic; https://github.com/aertslab/pycisTopic) to collect single-cell-level quality control statistics and filter barcodes starting from the PUMATAC fragments files. For quality control purposes, we considered all barcodes with at least ten unique fragments. We used the GRCh38 or mm10 BioMart^36^ gene annotation to calculate TSS enrichment. We followed the current ENCODE recommendations in calculating TSS enrichment by examining read depth in a 2,000-bp window on each side of the TSS (https://www.encodeproject.org/data-standards/terms/#enrichment). Cells were then filtered from barcodes using Otsu algorithm-defined thresholds on TSS enrichment and number of unique fragments per cell. In the first pass, we counted fragments in SCREEN regions^18^. This count matrix was then used to annotate cells with known cell types, after which consensus peaks could be called over all cell types present (see later). We then used this consensus peak set to recount the fragments and perform further downstream analyses in the second pass.

#### Donor identification (Freemuxlet in VSN/PUMATAC)

We used Freemuxlet to identify barcodes belonging to each of the two mixed individuals in each sample and simultaneously identify doublets on the basis of barcodes with mixed genotypes. We first ran a prefiltering step to filter the bam file for only the selected cells after the initial quality control. We then used the popscle suite of tools (https://github.com/statgen/popscle), first ‘dsc-pileup’ to quantify reads overlapping known variants and then Freemuxlet to call sample and doublet identity in each barcode. Freemuxlet requires a list of known variants in the genome along with their allele frequencies. To obtain this, we used the 1000 Genomes Phase 3 dataset^37^ and applied filtering steps to keep only SNPs with a minor allele frequency of at least 10%. This step was automated using VSN/PUMATAC^14^.

#### Doublet identification (Scrublet)

We used the Python implementation of Scrublet^19^ to identify doublets among the barcodes selected based on the fragments/regions count matrix during the initial quality control steps. Doublet thresholds for each sample were set manually, and doublets were removed for downstream analysis.

#### Sequencing depth downsampling (seqtk)

After the initial quality control steps to select cells, we identified the sample with the fewest number of reads per cell and used this sample as the reference to which the others were downsampled. After identifying the target number of reads per cell in the reference sample (ddS Bi3, with 40,796 reads per cell), each of the FASTQ files for the other samples were downsampled to that depth. Downsampling was performed with seqtk (https://github.com/lh3/seqtk, version 1.3-r106). The seed parameter (‘-s’) was set to the same value for all files to ensure that the reads remained paired across the paired-end and barcode files. Following downsampling of each FASTQ file, the mapping procedure was repeated to produce new downsampled fragments and bam files. This was repeated for 35,000, 30,000, 25,000, 20,000, 15,000, 10,000 and 5,000 reads per cell, and these FASTQ files were further processed as described earlier and later.

#### Cell-type identification (Seurat)

Label transfer was performed using an annotated PBMC reference dataset^21^ consisting of nine independent technology types and batches. We used Seurat (v4.0.3) to perform the label transfer steps in an R (v4.1.0) environment. Label transfer was performed using methods outlined in the Seurat vignettes and associated^22^. In brief, each of the nine already annotated PBMC reference datasets was compared pairwise to find cells that serve as anchors between them and then used to generate an integrated reference that minimized technical differences.

For the scATAC-seq data, we used this integrated PBMC reference to predict cell types. A gene activity matrix was first estimated, and label transfer was performed by assigning query cells based on the local neighborhood around each anchor in the integrated reference, with the highest scoring cell type being assigned. Following prediction of the scATAC-seq cell types, we refined these classifications by using the clusters identified in the scATAC-seq data. Clustering was first performed on cells with the Leiden algorithm using a high resolution to generate many fine-grained clusters. For each cluster, we then assigned a consensus cell-type identity to the entire cluster based on the majority cell type identified by label transfer. In this way, the ATAC-based clusters were labeled with the most likely cell type, while peak information was retained for later analysis. Where multiple clusters existed for one cell type, these were merged and used to generate cell-type-specific peak sets in downstream steps.

#### Two-pass dimensionality reduction (pycisTopic)

Fragments were first counted in ENCODE SCREEN regions to generate a preliminary count matrix. This count matrix was used to filter cells based on TSS enrichment and number of unique fragments. The SCREEN regions count matrix was then used to train cisTopic’s latent Dirichlet allocation models, and the model with an optimal number of topics was selected as described in ref. ^16^. Based on Seurat cell-type identification and high-resolution Leiden clustering, a consensus cell type was assigned to each cell. Cell-type-specific peaks (see later) were called based on these consensus cell types and aggregated into a new consensus peak set. The fragments were recounted in this new peak set to generate a consensus peak count matrix. This count matrix was used to retrain cisTopic’s latent Dirichlet allocation models, and an optimal model was chosen for the second pass and used to reduce the dimensionality of the data.

#### Consensus peak calling (pycisTopic)

In pyCisTopic, the ‘export_pseudobulk’ function was used to create cell-type-specific fragments and bigwig files using the consensus cell types. These were in turn used to generate cell-type-specific consensus peaks for each sample by recalling the subset of cells with MACS2 (ref. ^23^). Peak calling for quality control purposes was performed using MACS2 with settings specific to ATAC-seq experiments (‘genome_size = hs’, ‘shift = 73’, ‘ext_size = 146’ and ‘q_value = 0.01’). We used ‘Version 2’ of the ENCODE candidate *cis*-Regulatory Elements with blacklisted regions removed^38^ (Supplementary Fig. 1b). These regions were used after the quality control steps to create the cisTopic object, perform the first-pass clustering and obtain consensus peaks. Duplicate rates were calculated for each barcode by dividing the number of unique fragments by the total.

Cell-type-specific peak sets were generated for each sample. For each sample, the cell-type-specific peak sets were then merged into a final sample-specific consensus set. Each sample’s FRIP was calculated using these final consensus peaks. The consensus sets of all 47 samples were then merged into one master set, in which all data were counted to form the merged datasets.

#### Region overlap calculation (HOMER)

The mergePeaks function of the HOMER suite (v4.11)^39^ with default parameters (adding -d given -venn options) was used to find overlap between several region sets, notably the overlap between consensus DARs and peaks recovered in the merged cell-type fair set and in the individual cell-type fair sets.

#### DAR calling (pycisTopic)

A Wilcoxon rank-sum test was used to calculate significance of enrichment of regions (fold change) between each specified contrast using cisTopic’s imputed region accessibility based on cell topic and topic region probabilities. We contrasted cell types (type 1 versus all), technologies (cells from cell type 1 from technique A versus cells from cell type 1 from technique B) and donors (cells from cell type 1 from donor A versus cells from cell type 1 from donor B). Because high-quality samples produce many DARs that can skew the distribution of DAR enrichment scores to lower numbers, we chose to show the distributions of the top 2,000 DARs for each cell type contrast and the top 200 for male–female contrasts. For cell-type contrasts, differential accessibility was thresholded at a minimum of 1.5× fold change enrichment. For male/female contrasts, a minimum threshold of 1.2× fold change enrichment was used.

#### Transcription factor motif enrichment analysis (cisTarget)

Cell-type-specific and male/female-specific DARs were analyzed for transcription factor motif enrichment using cisTarget^24,25^ and standard parameters and settings for the human genome.

#### Price calculation and sequencing saturation

We calculated the price of a hypothetical 5,000-cell experiment based on US list prices for commercial methods and original manuscripts for open-source methods as follows. The 10x scATAC-seq v2 assay was quoted as $1,750 for 48 Next Gem Chip H (1000161), $930 for 96 indices (1000212) and $24,300 for 16 Chromium Next GEM Single Cell ATAC v2 (1000390) for a weighted total of $1,565 per lane. For the 10x multiome assay, the price was $1,750 for 48 Next Gem Chip J (1000234), $930 for 96 indices (1000215) and $44,760 for 16 Chromium Next GEM Single Cell Multiome ATAC + Gene Expression (1000283) for a weighted total of $2,843 per lane. For Bio-Rad ddSEQ SureCell ATAC, the price was $8,800 for a complete SureCell ATAC-Seq library prep kit (17004620), which accommodates eight samples. For s3-ATAC, the cost per plate is ~$200, and each plate can accommodate 1,440 cells. For HyDrop, the cost per run is ~$100 and can recover 8,000–10,000 cells.

Bio-Rad ddSEQ reports a doublet rate of 3.76% at a recovery of 5,000 cells (https://www.bio-rad.com/sites/default/files/webroot/web/pdf/lsr/literature/ATAC-Seq_Poster.pdf). 10x Chromium supports a recovery of up to 10,000 cells but at a doublet rate of 8%. At an expected doublet rate of 4%, 5,000 cells can be recovered (https://kb.10xgenomics.com/hc/en-us/articles/360001378811-What-is-the-maximum-number-of-cells-that-can-be-profiled-). HyDrop reports 6% doublets on 8,000 recovered cells ^12^. These three microfluidic methods use the same microfluidic concepts to encapsulate single cells, and doublet rates are similar when correcting for the number of cells recovered. We therefore reasoned that the most fair comparison would be to assume a recovery of 5,000 cells per 10x and ddSEQ lane or HyDrop run. For s3-ATAC, we assumed 1,440 cells per plate, for a total of 5,760 cells across four plates in s3-ATAC.

Full sequencing depth fragments files were subset to filtered cell barcodes (before doublet filtering and minimum TSS enrichment threshold, that is, only filtered by Otsu thresholding on minimum number of reads). We then subsampled these fragments files using a range of fractions and fitted a Michaelis–Menten kinetic model on the resulting duplication rate by the number of reads per cell. We defined the saturation sequencing depth at which each technology is expected to reach 50% duplicate fragments after sequencing.

#### scRNA-seq analysis (scanpy)

After aligning scRNA-seq data to the reference genome using CellRanger or CellRanger-arc, scanpy^40^ was used to calculate single-cell quality control metrics for each sample. True cells were filtered from noise using Otsu-derived cutoffs on minimum number of UMIs.

#### Public mouse brain data reanalysis

Public mouse scATAC-seq data were downloaded from the following sources: 10x Genomics scATAC-seq v1.0 on Chromium (https://s3-us-west-2.amazonaws.com/10x.files/samples/cell-atac/1.2.0/atac_v1_adult_brain_fresh_5k/atac_v1_adult_brain_fresh_5k_fastqs.tar), 10x Genomics scATAC-seq v1.1 on Chromium (https://s3-us-west-2.amazonaws.com/10x.files/samples/cell-atac/2.1.0/8k_mouse_cortex_ATACv1p1_nextgem_Chromium_X/8k_mouse_cortex_ATACv1p1_nextgem_Chromium_X_fastqs.tar), 10x Genomics scATAC-seq v2 on Chromium X (https://s3-us-west-2.amazonaws.com/10x.files/samples/cell-atac/2.1.0/8k_mouse_cortex_ATACv2_nextgem_Chromium_X/8k_mouse_cortex_ATACv2_nextgem_Chromium_X_fastqs.tar), 10x Genomics scATAC-seq v2 on Chromium (https://s3-us-west-2.amazonaws.com/10x.files/samples/cell-atac/2.1.0/8k_mouse_cortex_ATACv2_nextgem_Chromium_Controller/8k_mouse_cortex_ATACv2_nextgem_Chromium_Controller_fastqs.tar), 10x Genomics Multiome ATAC (https://s3-us-west-2.amazonaws.com/10x.files/samples/cell-arc/2.0.0/e18_mouse_brain_fresh_5k/e18_mouse_brain_fresh_5k_fastqs.tar), Bio-Rad ddSEQ (SRA accession number SRR14494477), s3-ATAC (SRA accession number SRX10841853) and HyDrop (SRA accession number PRJNA733185).

These data were reanalyzed in a similar manner as the PBMC datasets. Briefly, cells were filtered from noise as described above. All datasets were then downsampled to the highest common depth and further intervals of 5,000 reads per cell. These downsampled sets were realigned to the mm10 reference genome using PUMATAC and counted in the SCREEN regions. Cells were clustered, and per cluster peaks were called and aggregated into a consensus peak set per sample. Datasets were recounted in these consensus peak sets to produce the quality metrics shown in our manuscript.

### Reporting summary

Further information on research design is available in the Nature Portfolio Reporting Summary linked to this article.

## Online content

Any methods, additional references, Nature Portfolio reporting summaries, source data, extended data, supplementary information, acknowledgements, peer review information; details of author contributions and competing interests; and statements of data and code availability are available at 10.1038/s41587-023-01881-x.

### Supplementary information

Supplementary Information Supplementary Figs. 1–3.

Reporting Summary

Supplementary Table 1 Quality metrics for all individual samples (after downsampling to 40,000 reads per cell).

### Source data

Source Data Fig. 1 Source data for Fig. 1.

Source Data Fig. 2 Source data for Fig. 2.

Source Data Fig. 3 Source data for Fig. 3.

Source Data Fig. 4 Source data for Fig. 4.

Source Data Extended Data Fig. 1 Source data for Extended Data Fig. 1.

**Extended Data Fig. 4:**
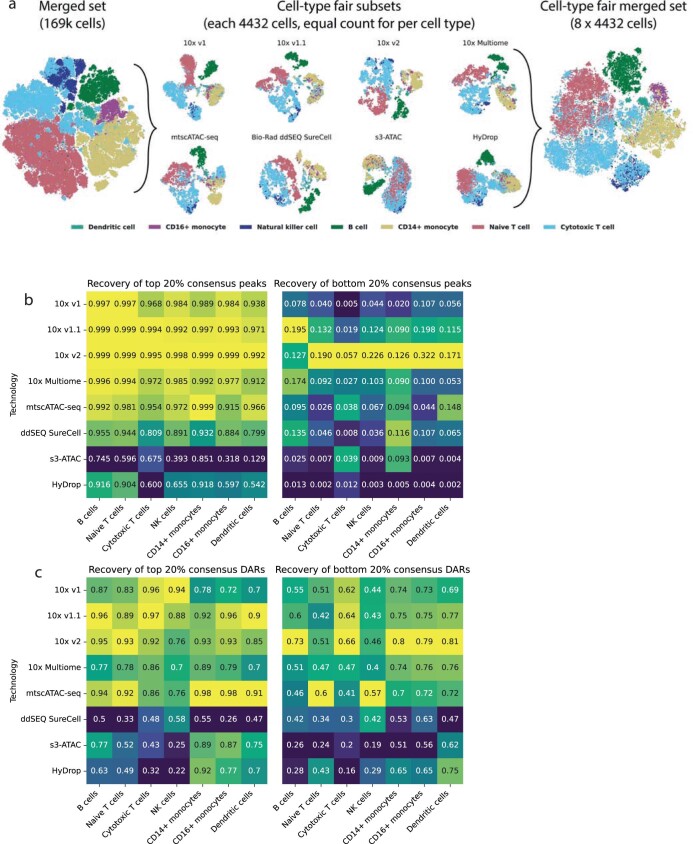
Cell-type fair subsetting and peak/DAR calculation. **a**, Diagram showing cell-type fair subsetting strategy. **b**, Heatmap showing the fraction of cell-type-fair peaks recovered by the individual technologies’ subsets, separated by cell type. **c**, Heatmap showing the fraction of cell-type-fair DARs recovered by the individual technologies’ subsets, separated by cell type. Source data

**Extended Data Fig. 5:**
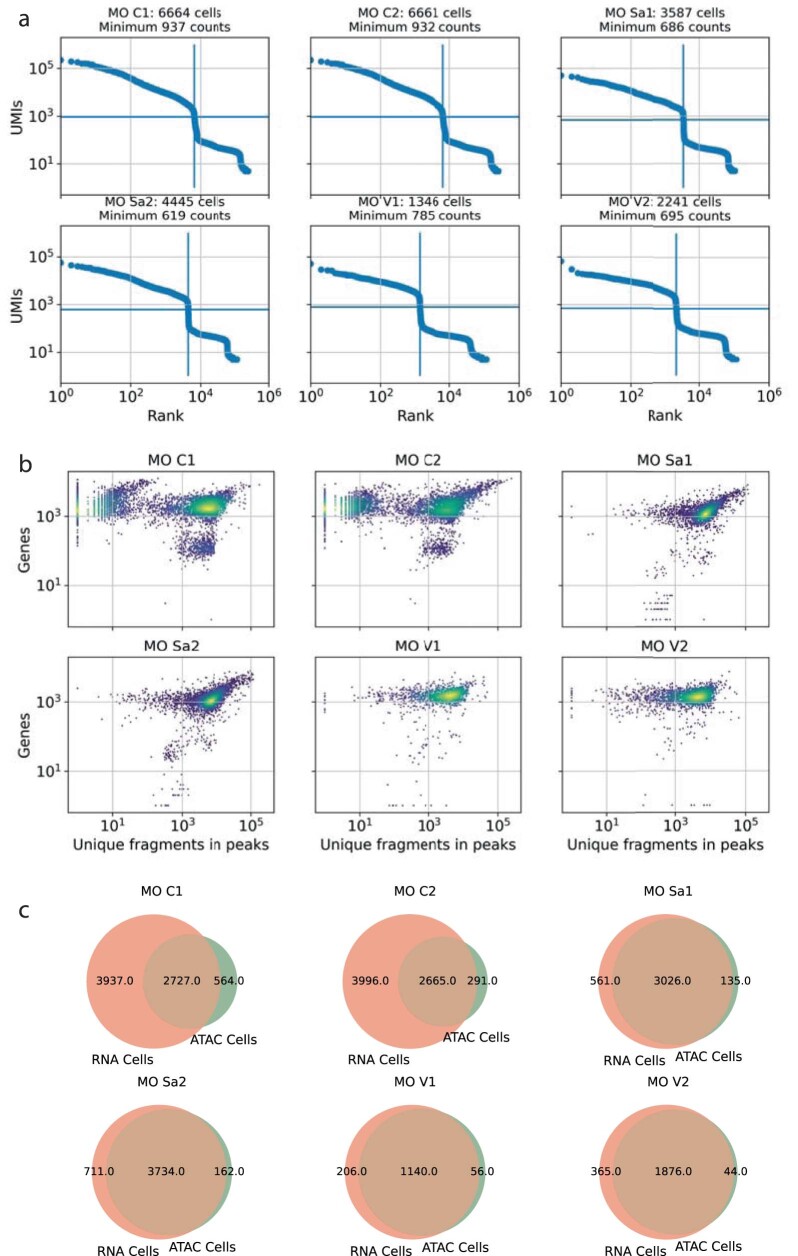
10x multiome RNA performance. **a**, Knee plots of RNA component of 10x multiome samples. Minimum UMI thresholds were automatically defined using Otsu’s algorithm. **b**, Scatterplots of number of genes versus unique fragments in peaks detected in the same multiome cells. **c**, Venn diagrams showing overlap between cell barcodes identified as cells based on ATAC or RNA components.

Source Data Extended Data Fig. 4 Source data for Extended Data Fig. 4.

**Extended Data Fig. 6:**
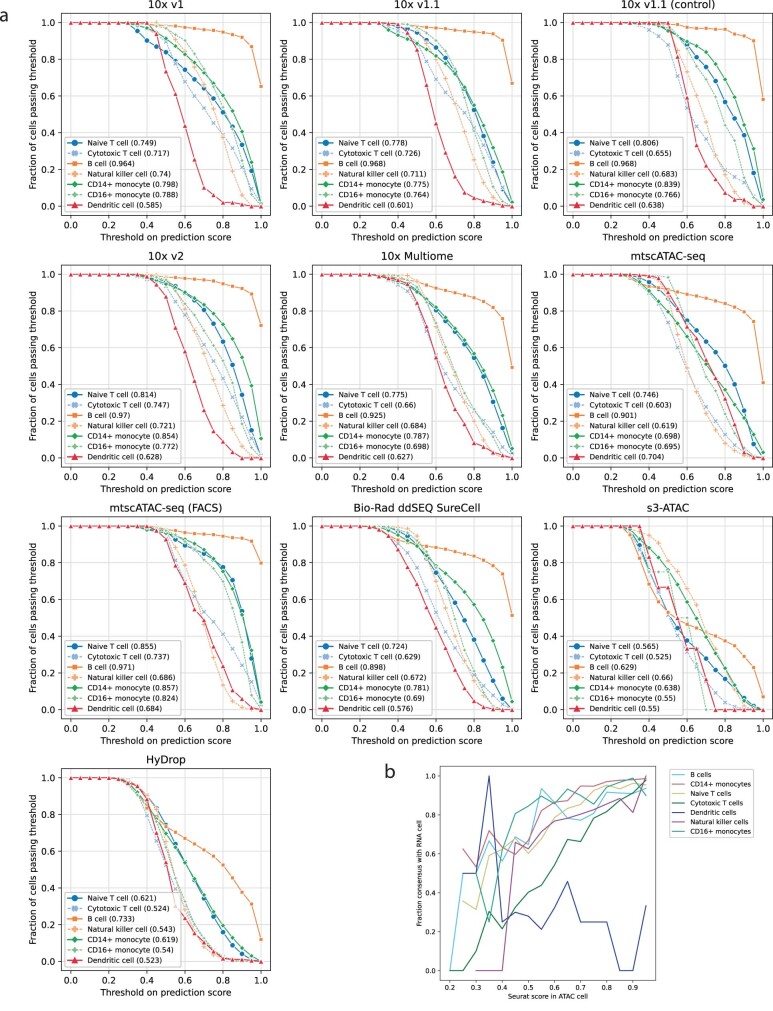
Threshold prediction curves for Seurat label transfer scores. **a**, Lineplots showing fraction of reads passing the threshold with increasing thresholds. Area under curve (AUC) values for each cell type in figure legends. **b**, Lineplot showing label transfer agreement between RNA and ATAC modalities of 10x Multiome samples on y axis, and Seurat scores in the ATAC cell. Source data

Source Data Extended Data Fig. 6 Source data for Extended Data Fig. 6.

**Extended Data Fig. 7:**
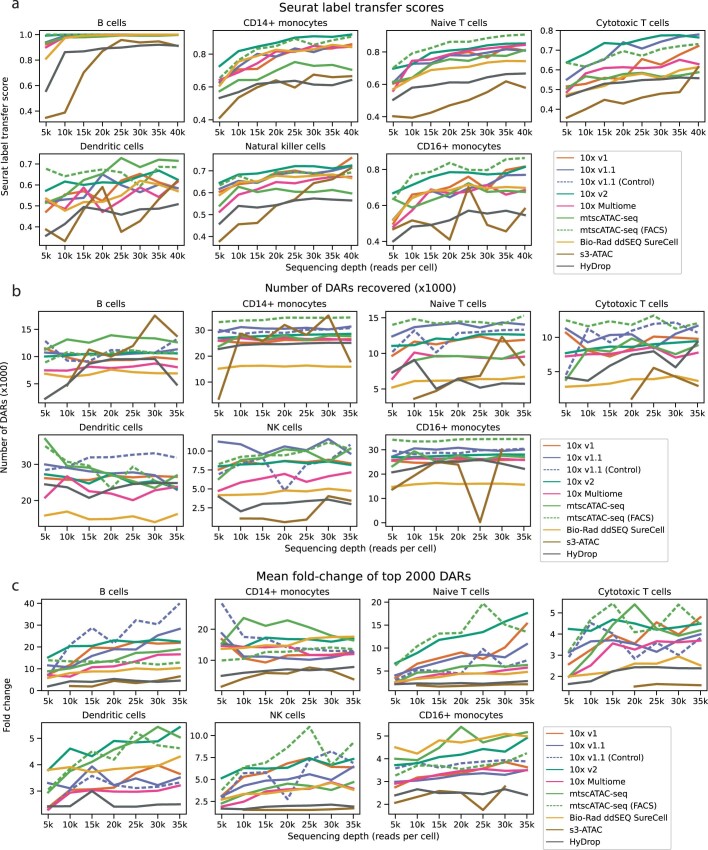
Effect of sequencing depth on cell type annotation and DAR calling. For these figures, all samples were downsampled to 35k, 30k, … reads per cell, and de-novo annotated with Seurat. **a**, Dependency of Seurat label transfer scores on sequencing depth. **b**, Dependency of number of DARs detected in each cell type on sequencing depth. **c**, Dependency of mean fold-change enrichment of top 2000 DARs detected in each cell type on sequencing depth. Source data

Source Data Extended Data Fig. 7 Source data for Extended Data Fig. 7.

**Extended Data Fig. 8:**
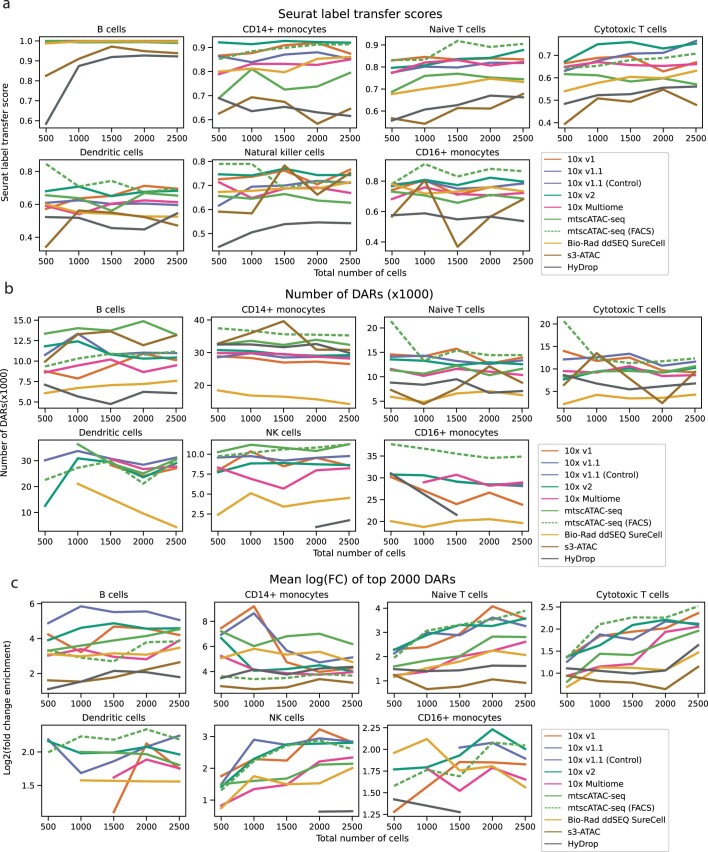
Effect of number of cells on cell type annotation and DAR calling. For these figures, all samples with at least 2,500 cells were subset to 2,500, 2,000, … random cells, and de-novo annotated with Seurat. **a**, Dependency of Seurat label transfer scores on total number of cells. **b**, Dependency of number of DARs detected in each cell type on total number of cells. **c**, Dependency of mean log2 fold-change enrichment of top 2,000 DARs detected in each cell type on total number of cells. Source data

Source Data Extended Data Fig. 8 Source data for Extended Data Fig. 8.

**Extended Data Fig. 9:**
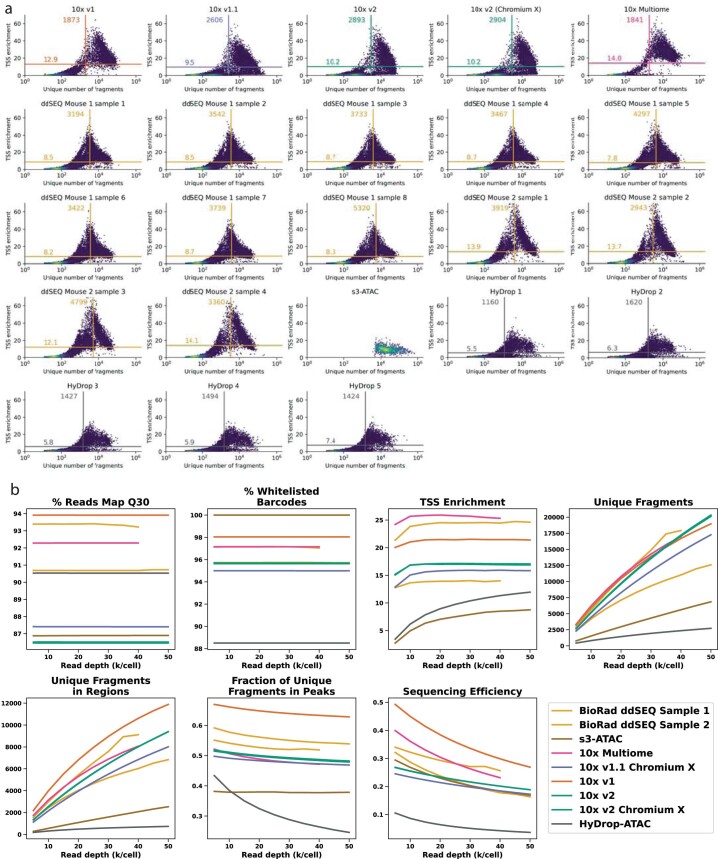
Public mouse cortex data reanalysis statistics. **a**, TSS enrichment and unique number of fragments distributions for public mouse datasets reanalysed using PUMATAC. **b**, Dependency of basic quality metrics on sequencing depth across techniques. Source data

Source Data Extended Data Fig. 9 Source data for Extended Data Fig. 9.

**Extended Data Fig. 10:**
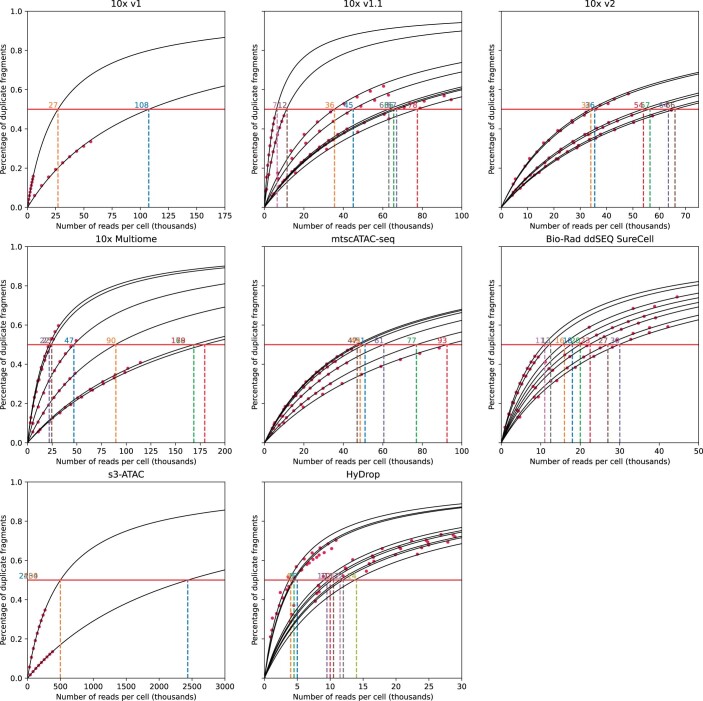
Duplication rate in fragments files. X-axis shows the number of reads per cell (thousands), y-axis shows the percentage of fragments in cells that are duplicates. Red dots indicate data points sampled from fragments files, black lines are a fitted Michaelis-Menten curve. Red line indicates a 50% duplicate rate. Source data

Source Data Extended Data Fig. 10 Source data for Extended Data Fig. 10.

## Data Availability

Single-cell ATAC accessibility and gene expression data can be viewed at https://scope.aertslab.org/#/scATAC-seq_Benchmark/scATAC-seq_Benchmark. Single-cell ATAC coverage bigwigs and DAR/peak BEDs can be downloaded at https://ucsctracks.aertslab.org/papers/scatac_benchmark/ and viewed using University of California Santa Cruz’s custom track hub. Sequencing data, fragments files and count matrices are freely available at Gene Expression Omnibus under accession number GSE194028 (ref. ^41^). Summary quality metrics for all samples can be found in Supplementary Table 1. Source data are provided with this paper.

## Code availability

All data analysis scripts can be found at https://github.com/aertslab/scATAC-seq_benchmark ref. ^42^. PUMATAC can be found at https://github.com/aertslab/PUMATAC.
