# Supplementary material for: Systematic benchmarking of single-cell ATAC-sequencing protocols
Source: Nat Biotechnol. 2023 Aug 3;42(6):916–26. doi: 10.1038/s41587-023-01881-x (PMC11180611; doi:10.1038/s41587-023-01881-x)
Supplement: Supplementary file 1 — Supplementary Figs. 1–3. [file 41587_2023_1881_MOESM1_ESM.pdf]

nature biotechnology

Article

https://doi.org/10.1038/s41587-023-01881-x

Systematic benchmarking of single-cell ATAC-sequencing protocols

In the format provided by the authors and unedited

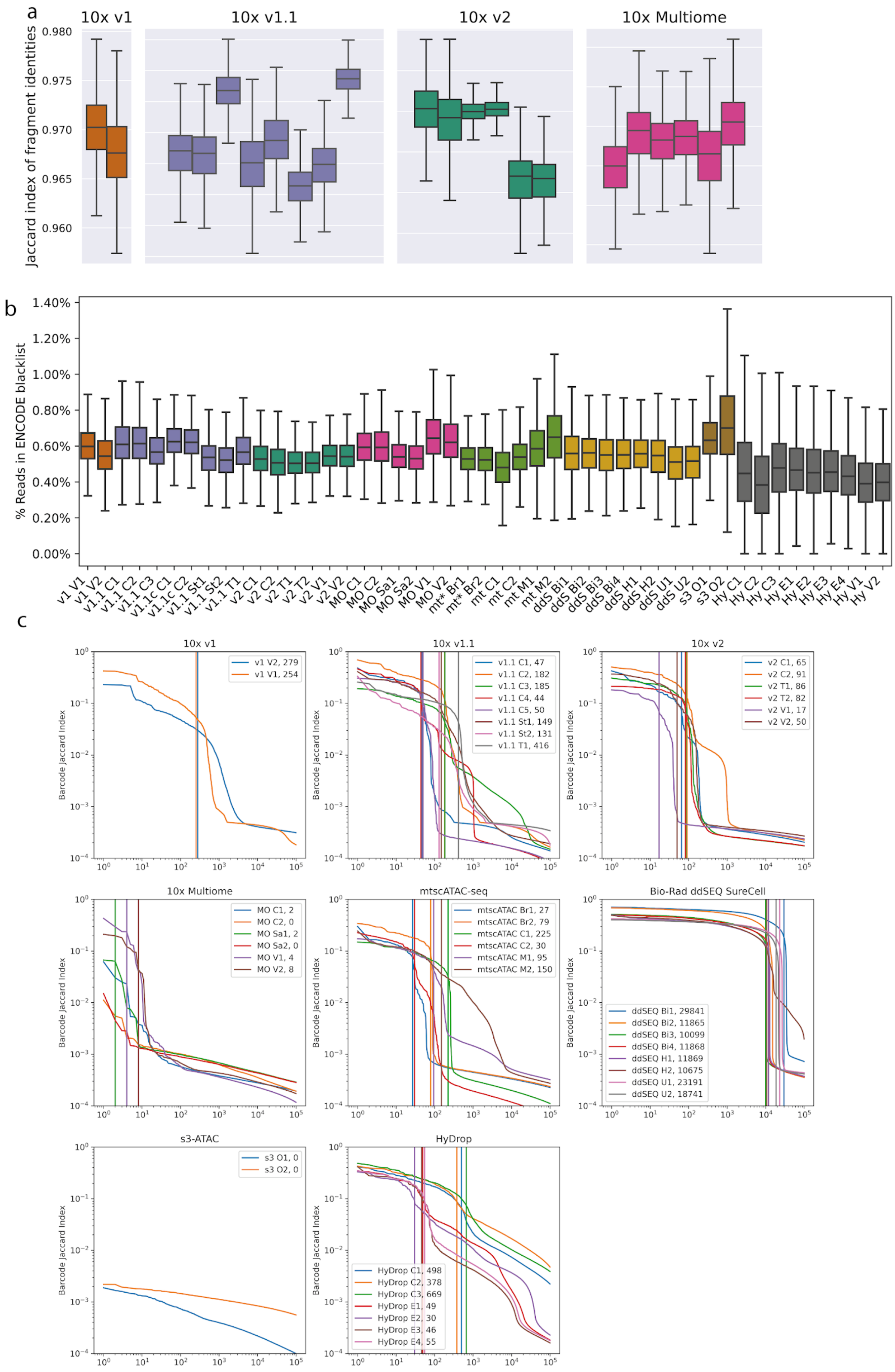

Supplementary Figure 1. Comparison of PUMATAC and CellRanger fragments and barcode multiplet statistics. a. Jaccard index of fragment identity (barcode + begin and end position) overlap between fragments files generated by

10x CellRanger and PUMATAC. Each datapoint underlying the boxplot is a barcode, and the Jaccard value is the overlap in fragment identity with the same barcode in the CellRanger fragments file. n = 1,614,073,154 fragments examined over 16 independent experiments. b. Fraction of reads aligning to ENCODE blacklist. n = 3,627,309,389 fragments examined over 47 independent experiments. Median and quartiles indicated by box, whiskers indicate 1.5x interquartile range, minima/maxima/center not indicated. c. Kneeplots of barcode multiplet Jaccard index calculated by PUMATAC. Jaccard index is calculated as the fractional overlap in fragment identity between pairs of barcodes.

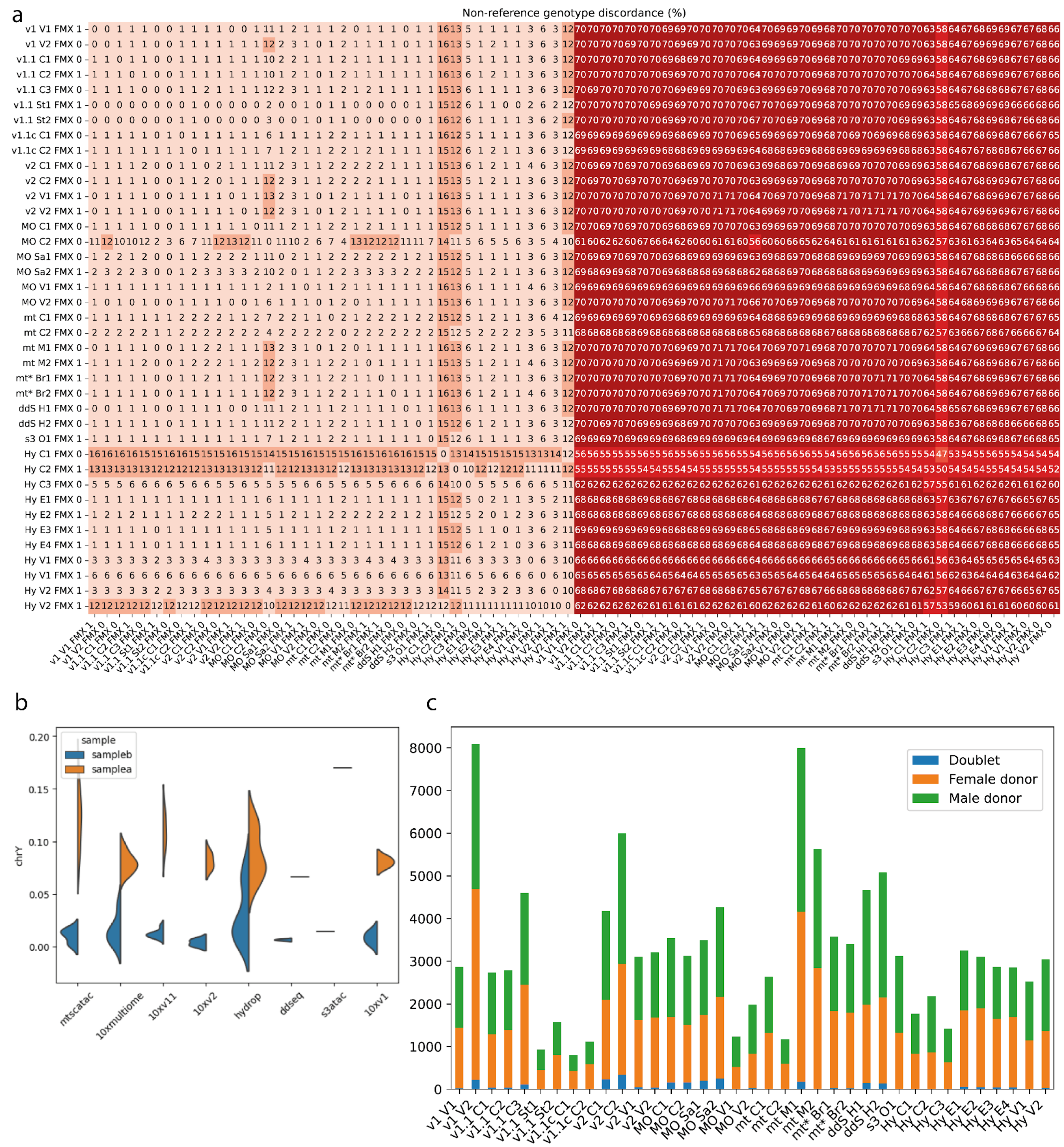

Supplementary Figure 2. Donor assignment and identification. a. Pairwise genotype concordance rate between donor 0 and donor 1 calculated by Freemuxlet. b. Fraction of fragments mapping to chromosome X in sample A and B, split by technique. c. Number of cells assigned to each donor in each experiment.

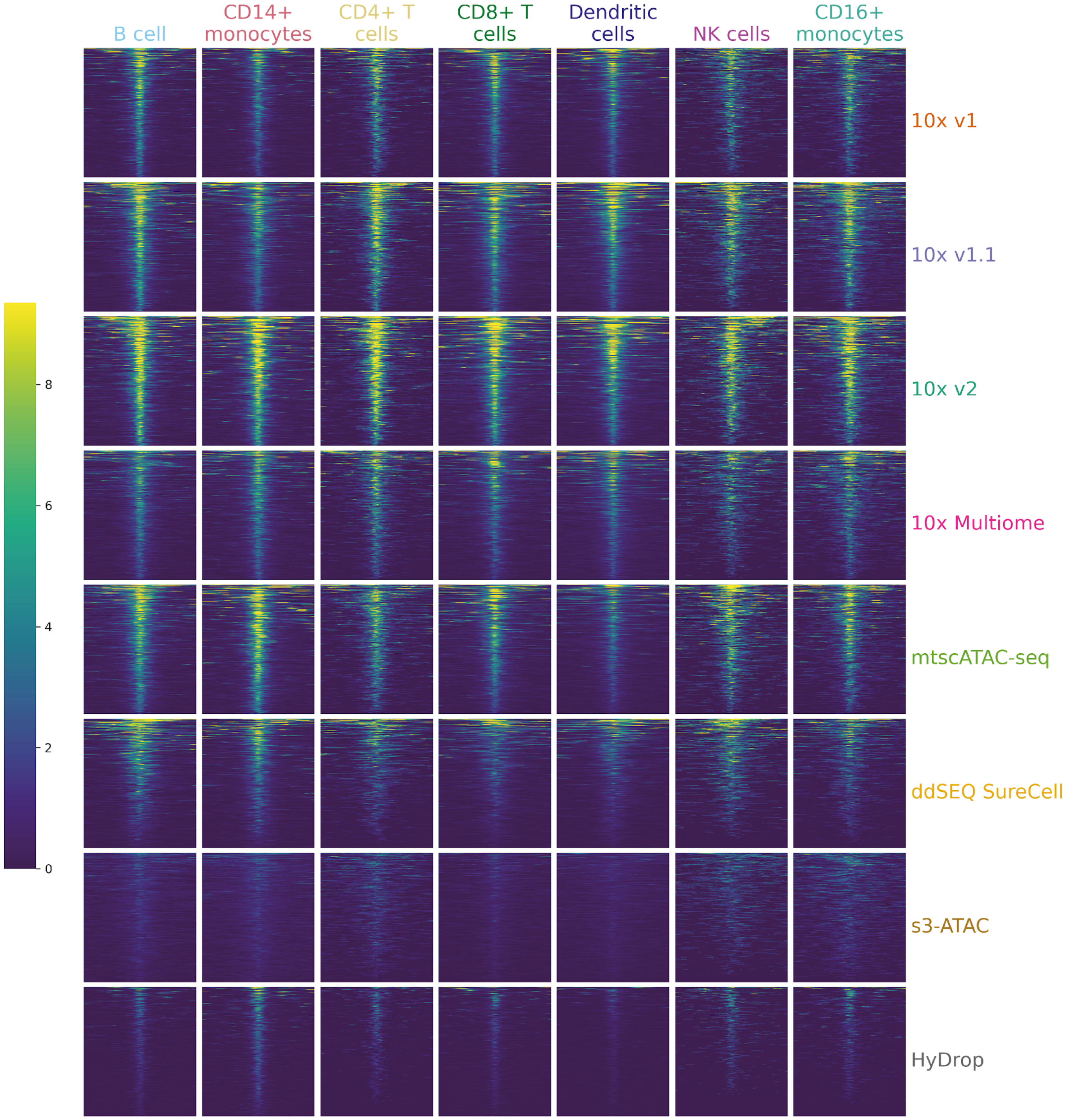

Supplementary Figure 3. Genomic coverage heatmaps in a 2500 bp window centered around cell-type fair DARs. For each cell type, the strongest 2,000 DARs detected in the cell-type fair merged set of 8 x 4,432 cells are taken, and the genomic coverage within cells of that cell type in each technique's cell-type fair subset, within a 2500 bp window centered around each DAR is drawn. In each column, the color intensity is scaled to that column's minimum and maximum value. Cell counts across techniques are equal within a single cell type, so coverage differences between techniques can be compared without accounting for cell counts.
