# Supplementary material for: Systematic benchmarking of single-cell ATAC-sequencing protocols
Source: Nat Biotechnol. 2023 Aug 3;42(6):916–26. doi: 10.1038/s41587-023-01881-x (PMC11180611; doi:10.1038/s41587-023-01881-x)
Supplement: Supplementary file 2 — Reporting Summary [file 41587_2023_1881_MOESM2_ESM.pdf]

nature portfolio

Corresponding author(s): Holger Heyn
Stein Aerts

Last updated by author(s): Jun 13, 2023

Reporting Summary

Nature Portfolio wishes to improve the reproducibility of the work that we publish. This form provides structure for consistency and transparency in reporting. For further information on Nature Portfolio policies, see our Editorial Policies and the Editorial Policy Checklist.

Statistics

For all statistical analyses, confirm that the following items are present in the figure legend, table legend, main text, or Methods section.

n/a	Confirmed	
☐	☒	The exact sample size (*n*) for each experimental group/condition, given as a discrete number and unit of measurement
☐	☒	A statement on whether measurements were taken from distinct samples or whether the same sample was measured repeatedly
☐	☒	The statistical test(s) used AND whether they are one- or two-sided<br>*Only common tests should be described solely by name; describe more complex techniques in the Methods section.*
☒	☐	A description of all covariates tested
☒	☐	A description of any assumptions or corrections, such as tests of normality and adjustment for multiple comparisons
☐	☒	A full description of the statistical parameters including central tendency (e.g. means) or other basic estimates (e.g. regression coefficient) AND variation (e.g. standard deviation) or associated estimates of uncertainty (e.g. confidence intervals)
☒	☐	For null hypothesis testing, the test statistic (e.g. *F*, *t*, *r*) with confidence intervals, effect sizes, degrees of freedom and *P* value noted<br>*Give P values as exact values whenever suitable.*
☒	☐	For Bayesian analysis, information on the choice of priors and Markov chain Monte Carlo settings
☒	☐	For hierarchical and complex designs, identification of the appropriate level for tests and full reporting of outcomes
☒	☐	Estimates of effect sizes (e.g. Cohen's *d*, Pearson's *r*), indicating how they were calculated

Our web collection on statistics for biologists contains articles on many of the points above.

Software and code

Policy information about availability of computer code

Data collection

No code was used to collect in-silico data for this study.

Data analysis

All software used, including versions, and all scripts used are described in detail in our github repo: https://github.com/aertslab/scATAC-seq_benchmark

We use PUMATAC, our own genome alignment pipeline (previously called VSN-ATAC), which can be found here: https://github.com/aertslab/PUMATAC

PUMATAC uses the following packages:
seqtk version 1.3-r106
popscle (no version info available on github: https://github.com/statgen/popscle, last updated before we started this work)
bwa mem2 (no version info available on github: https://github.com/bwa-mem2/bwa-mem2 last updated before we started this work)

We use the following Python packages with python 3.8.15:

MACS2	2.2.7.1
matplotlib	3.6.1
numpy	1.23.4
pandas	1.5.0
polars	0.14.18

ray	2.0.0
scanpy	1.9.1
scikit-image	0.19.3
scikit-learn	1.1.2
scipy	1.9.2
scrublet	0.2.3
seaborn	0.12.0
pycisTopic	1.0.1.dev21+g8aa75d8.d20221014

We also use R version 4.1.0. We only use Seurat v4.0.3. and its dependencies.

We also use the web version of cisTarget, which can be found here: https://gbiomed.kuleuven.be/apps/lcb/i-cisTarget/
For PUMATAC, Nextflow 21.04.03 was used.
We used HOMER (v4.11).

For manuscripts utilizing custom algorithms or software that are central to the research but not yet described in published literature, software must be made available to editors and reviewers. We strongly encourage code deposition in a community repository (e.g. GitHub). See the Nature Portfolio guidelines for submitting code & software for further information.

Data

Policy information about availability of data

All manuscripts must include a data availability statement. This statement should provide the following information, where applicable:

- Accession codes, unique identifiers, or web links for publicly available datasets
- A description of any restrictions on data availability
- For clinical datasets or third party data, please ensure that the statement adheres to our policy

Single-cell ATAC accessibility and gene expression data can be viewed at https://scope.aertslab.org/#/scATAC-seq_Benchmark/scATAC-seq_Benchmark. Single-cell ATAC coverage bigwigs and DAR/peak BEDs can be downloaded at https://ucsctracks.aertslab.org/papers/scatac_benchmark/ and viewed using UCSC's custom track hub. Sequencing data, fragments files and count matrices are freely available at GEO, under accession number GSE194028 (26). Summary quality metrics for all samples can be found in Supplementary table 1.

Research involving human participants, their data, or biological material

Policy information about studies with human participants or human data. See also policy information about sex, gender (identity/presentation), and sexual orientation and race, ethnicity and racism.

Reporting on sex and gender	Not relevant to our study, since we do not attempt to draw biological conclusions pertaining to sex or gender. Rather, our benchmark uses differences in chromatin accessibility between our male and female donor to validate each benchmarked technology's ability to resolve such subtle differences. The terms "sex" and "gender" have not been confused in our manuscript.
Reporting on race, ethnicity, or other socially relevant groupings	Not relevant to our study, since we do not attempt to draw biological conclusions pertaining to sex or gender. Rather, our benchmark uses differences in chromatin accessibility between our male and female donor to validate each benchmarked technology's ability to resolve such subtle differences.
Population characteristics	Not relevant to our study, this information is unknown (2 anonymous, commercial PBMC donors were used).
Recruitment	PBMC from 2 donors were purchased from AllCells.
Ethics oversight	Only commercially available PBMC were used (AllCells)

Note that full information on the approval of the study protocol must also be provided in the manuscript.

Field-specific reporting

Please select the one below that is the best fit for your research. If you are not sure, read the appropriate sections before making your selection.

☒ Life sciences ☐ Behavioural & social sciences ☐ Ecological, evolutionary & environmental sciences

For a reference copy of the document with all sections, see nature.com/documents/nr-reporting-summary-flat.pdf

Life sciences study design

All studies must disclose on these points even when the disclosure is negative.

Sample size	No sample size calculation was performed. Each center performed as many experimental replicates as feasible, and we aimed for at least 3 sets of 2 replicates per technology. This condition was achieved for all techniques except s3-ATAC (due to the low number of labs capable of performing this protocol) and 10x v1.0 (since this kit has been surpassed by v1.1 and lately v2, and has been discontinued). We chose to perform replicates to assess technical variability across experiments and to perform triplicates across center where a higher variance in performance was expected. These sample sizes are sufficient for our study, since the Games-Howell test (which is more conservative than

Bonferroni or Tukey tests) shows significance for the effects we discuss in our manuscript. Ideally we would have liked to include more s3-ATAC samples, but the technique proved less robust/sensitive than we thought at the beginning of the study, so it was difficult to find more labs both capable of and willing to perform this technology.

Data exclusions	No data generated for this study were excluded. Analysis scripts contain traces of PBMC Bio-Rad ddSEQ scATAC-seq samples that were generated at CNAG, but not for the purpose of this study. These samples were candidate for inclusion in the study, but were not kept as their cell counts were too low to qualify (700 cells only, and the design of our benchmark aimed for 3000 cells per sample).
Replication	A total of 47 experiments were performed for this study. Across all samples, we performed 6 technical replicates for 10x v2, 2 for 10xv1, 6 for 10x v1.1 + 2 control runs with different sample preparation, 6 for 10x Multiome, 4 for mtscATAC-seq + 2 mtscATAC-seq + FACS experiments, 8 ddSEQ SureCell experiments, 2 s3-ATAC, and 9 for HyDrop. Sample preparation was standardized where possible (following the 10x demonstated protocols for PBMC thawing and nuclei extraction, for example). We also performed control experiments for samples that did not allow for this sample preparation (e.g Bio-Rad ddSEQ and s3-ATAC samples)
Randomization	We did not randomize the techniques across centres, as not all centres have the capabilities to perform all techniques. The original authors of each technique were involved for all techniques to ensure that the most qualified operators were performing experiments, and we attempted to incorporate as many techniques as possible across all the participating centres. For all techniques except s3-ATAC and 10x v1, at least 3 different centres were involved in performing experiments.
Blinding	Blinding was not possible or practical for our study, since the technologies contain specific signatures in their data (e.g. barcode location and whitelist). In theory, samples generated from the same technology, but in different centres, could have been blinded to the lead researcher performing all analyses (FDR), but this would not be effective as all data was analysed on a running basis, and samples were added at several time points during analysis.

Reporting for specific materials, systems and methods

We require information from authors about some types of materials, experimental systems and methods used in many studies. Here, indicate whether each material, system or method listed is relevant to your study. If you are not sure if a list item applies to your research, read the appropriate section before selecting a response.

Materials & experimental systems

n/a	Involved in the study	
☒	☐	Antibodies
☒	☐	Eukaryotic cell lines
☒	☐	Palaeontology and archaeology
☒	☐	Animals and other organisms
☒	☐	Clinical data
☒	☐	Dual use research of concern
☒	☐	Plants

Methods

n/a	Involved in the study	
☒	☐	ChIP-seq
☒	☐	Flow cytometry
☒	☐	MRI-based neuroimaging
